# Survey of temporal coding of sensory information

**DOI:** 10.3389/fncom.2025.1571109

**Published:** 2025-07-02

**Authors:** Peter Cariani, Janet M. Baker

**Affiliations:** ^1^Hearing Research Center, Boston University, Boston, MA, United States; ^2^Harvard Medical School, Boston, MA, United States; ^3^Massachusetts Institute of Technology, Cambridge, MA, United States

**Keywords:** multiplexing, phase-locking, neural coding, sensory coding, spike latency, perception

## Abstract

Here we present evidence for the ubiquity of fine spike timing and temporal coding broadly observed across sensory systems and widely conserved across diverse phyla, spanning invertebrates and vertebrates. A taxonomy of basic neural coding types includes channel activation patterns, temporal patterns of spikes, and patterns of spike latencies. Various examples and types of combination temporal-channel codes are discussed, including firing sequence codes. Multiplexing of temporal codes and mixed channel-temporal codes are considered. Neurophysiological and perceptual evidence for temporal coding in many sensory modalities is surveyed: audition, mechanoreception, electroreception, vision, gustation, olfaction, cutaneous senses, proprioception, and the vestibular sense. Precise phase-locked, phase-triggered, and spike latency codes can be found in many sensory systems. Temporal resolutions on millisecond and submillisecond scales are common. General correlation-based representations and operations are discussed. In almost every modality, there is some role for temporal coding, often in surprising places, such as color vision and taste. More investigations into temporal coding are well-warranted.

## 1 Introduction

“If everyone else is looking down, look up or in a different direction. You will be surprised at what you will find.” Grote Reber, inscription at Green Bank Observatory, West Virginia.

“Look where I am pointing, don’t bite my finger.” Warren McCulloch (Papert, 1965).

This is a survey of temporal codes with pointers to evidence for them in sensory systems. Evidence for temporal coding of sensory distinctions can be found in nearly every sensory modality. This paper surveys this body of evidence and attempts to bring it together into a systematic, unified theoretical perspective. Temporal codes may well turn out to be as fundamental as rate-based channel codes.

First, the paper takes up the problem of neural coding and outlines basic types of temporal codes. The second half of the paper presents an overview of some of the neurophysiological and psychological evidence that supports various possible roles for temporal codes in major sensory modalities, updating and expanding three previous papers on the subject (Cariani, 1995, 2001b; Cariani, 2004).

## 2 The neural coding problem

Arguably, the specific nature of neural coding in the brain is the most fundamental unsolved problem in neuroscience because it determines how we think about neural systems, both natural and artificial. The neural coding problem entails identifying which specific aspects of neural activity convey those informational distinctions on which brain functions depend (Uttal, 1972; Gerstner et al., 1997; Rieke et al., 1997; Gerstner, 1999). How do collections of spike trains at different brain loci encode the various types of information that brains handle?

Despite enormous advances, because we have yet to solve the neural coding problem for most brain subsystems, neuroscience is still in a phase similar to biology before the elucidation of the genetic code. Without knowledge of the basic signals of the system, what aspects of neural activity subserve what functions, it is difficult to understand how brains work in terms of specific neurocompuational mechanisms, let alone how their informational functions go awry or how such dysfunctions can be remedied.

There are two main approaches to neural coding. Neural coding can be approached from a purely mathematical, Shannonian information-theoretic perspective (how much information is conveyed through some set of alternative signals through a specified transmission channel, (MacKay and McCulloch, 1952; Borst and Theunissen, 1999; Dimitrov et al., 2011) or from a function-based perspective (what is the relation of a set of signals to the informational functions of a system).

The information-theoretic approach often avoids specific considerations of function, relying instead on general assumptions involving optimally efficient coding (Barlow, 1961; Barlow, 1995) or optimal use of potentially available information by central processors (MacKay and McCulloch, 1952; Siebert, 1970; Heinz et al., 2001). Using such empirical and theoretical optimal use estimates, informational capacities of temporal codies often exceed those for rate-channel codes by an order of magnitude or more. On its face, the efficient coding principle does not comport with neural populations that typically have high average spontaneous firing rates (e.g., >50 spikes/s), such as auditory and vestibular nerves. Although the optimum central processor assumption is useful in ruling out prospective codes that have insufficient informational capacity to account for perceptual acuities, once adequacy has been established, higher transmission rates (e.g., MacKay and McCulloch, 1952) do not *a priori* favor one code over another. This is because we often do not know what are the primary operational measures, e.g., precision, accuracy, robustness, reliability, fail-safe, damage-resistance/survivability, for which a central processor might be optimized, as well as structural and developmental constraints on its biological realization. When there exists a surfeit of informational capacity, it is also relatively easy to pool information in order to achieve higher levels of robustness and reliability at the expense of high precision and accuracy.

In contrast, the function-based conception of neural coding focuses on functional meaning in seeking to find pervasive correspondences with perception and behavior. Instead of optimality of informational efficiency, in order to operate in a robust manner in wide ranges of noisy and unpredictable contexts, a good deal of redundancy and suboptimal pooling of information is perhaps to be expected. Optimality of predictive accuracy under a wide range of unpredictable and novel conditions is distinct from efficient neural coding.

In the spirit of Gregory Bateson (“The difference that makes a difference”), distinctions are differences that make a difference in terms of internal functional states and consequent behaviors. In the functional view, the framework adopted here, the neural coding problem involves identifying those systematic differences in neural activity that subserve functional differences. Neural coding is thus the problem of reverse-engineering the functional, “signals of the system.”

The neural coding problem concerns both relations of stimuli to neural activity (“encoding”, in what forms information available to the system may take) and relations of neural activity to behavior (“decoding,” how the system uses this information to effect appropriate behaviors)(Shimazaki, 2025).

For several reasons this paper focuses on temporal coding of sensory distinctions, i.e., how “sensory information” is encoded, as opposed to those codes that support central neural representations (e.g., in cognition, emotion, motivation, memory traces, and motor programs). First, the specific nature of neural coding of specific basic sensory and perceptual attributes is generally much better understood than those codes involved in more central functional states. Part of this is because sensory neural states and their associated individual attributes tend to be simpler and more easily identified than their more complex, multi-attribute and multi-modal cognitive representations.

Whereas neural response patterns at stations near sensory surfaces are mainly driven by external stimuli and hence are more easily correlated with both stimulus structure and associated perceptual attributes, more central distinctions can involve other brain systems whose neural responses are contingent on past history and current motivational, emotional, cognitive, and mnemonic states. Second, large numbers of discriminable stimulus states means that coding schemes can be tested with high precision, and some assessment can be made of the adequacy of a candidate code in accounting for perceptual acuities. When experiments rely on 1-bit detections or discriminations between or recognitions of small sets of stimuli, it can be difficult to determine whether some specific code or another is being used by the brain for that task. Third, perceptual invariances in which different stimuli and stimulus conditions evoke the same perceptual attributes afford a means of testing how well the general behavior of a particular neural code or representation resembles that of the perceptual system it is presumed to serve.

Neural candidate codes are those putative coding schemes which cannot be ruled out of hand and for which there exist plausible correlations with observed (here, sensory) functions. Definitive establishment of a code requires positive demonstration of a causal (in addition to a correlative) relationship between so-coded neural states and specific sensory distinctions (Mountcastle, 1988; Mountcastle, 1993; Cariani, 1995, 1999; Jazayeri and Afraz, 2017; Panzeri et al., 2017).

Neuropsychological models that successfully predict perceptual judgments for specific stimuli on the basis of observed or biologically-realistic simulated neural responses can provide positive demonstration that some specified code is used by some nervous system for some particular functional end. Electrical stimulation that produces specific patterns of neural activity and evoked percepts consistent with neural coding hypotheses provide additional powerful evidence for those neural codes. Appropriately temporally patterned electrical stimulation has been found to produce percepts related to flutter-vibration, auditory pitch and speech perception (via cochlear implants), color, taste, and pain. More recently, optical stimulation has been used in a similar manner to test coding hypotheses, e.g., (Chong et al., 2020; Bagur et al., 2025).

Although we are generally skeptical of automatic assumptions of optimal efficiencies and ideal observers, information-theoretic models *are* quite useful in ruling out candidate codes if they find that a prospective code lacks enough informational capacity to account for the acuity or robustness of some perceptual capability (Jacobs et al., 2009). These analyses can also be quite useful in engineering contexts by suggesting new alternative coding schemes that can yield superior precision, robustness, information capacity, and efficiency in artificial devices (section “13 Design of artificial systems”).

## 3 Temporal codes

### 3.1 What are temporal codes?

Temporal codes are those neural codes in which information is carried by timings of receptor activations (Rucci et al., 2018). Temporal codes are based on temporal patterns of neuronal spiking responses rather than on which specific neurons or neuronal populations are differentially activated (“channel” or “rate-place” codes). Whereas temporal codes rely on temporal relations between spikes, channel codes rely on activation profiles amongst selective, tuned receptive elements. In the footsteps of Johannes Mueller’s theory of specific nerve energies, channel coding assumes that the particular neurons that “respond”, i.e., are most excited in some way, encode the sensory quality that is perceived. Following Helmholtz and Adrian, channel coding, specifically rate-place coding, has been the dominant, default assumption in neuroscience.

However, temporal coding has always constituted an alternative to rate-place coding, with an early lineage in acoustics and auditory theory. In the 19th century it runs from Seeboeck’s siren to Rutherford’s “telephone theory” of audition, in the 20th it begins with the temporal spike pattern theories of pitch of Troland (1930) and Wever (1949) (Wever and Bray, 1937; Boring, 1942; de Cheveigné, 2005). Notably, inspired by radio heterodyning, Troland also proposed a temporal, “nerve current modulation” theory of color (Troland, 1921, 1930), p. 200–202) in which temporal patterns related to color would be multiplexed with those encoding other visual attributes. Subsequent auditory models of Jeffress (1948) and Licklider (1951a) 1959 combined temporal and channel-coding principles. Interest in the function-oriented neural coding problem has waxed and waned since, with peaks in the 1960’s (Mountcastle, 1967; Perkell and Bullock, 1968; Perkell, 1970), early 1970’s (Uttal, 1972; Uttal, 1973), and the 1990’s (Wasserman, 1992; Carr, 1993b; Cariani, 1995; Covey et al., 1995; Theunissen and Miller, 1995; Rieke et al., 1997; Lestienne, 1999; Cariani, 2001b; Panzeri et al., 2009).

The temporal patterns that form the basis for temporal codes can exist at the levels of spike trains of single neurons, ensembles, subpopulations, or whole populations. The temporal patterns can involve differences of characteristic time intervals between spikes, as in interspike interval codes (e.g., 10 vs. 11 ms intervals in auditory nerve fibers), or in spike arrival times (e.g., relative sub-millisecond differences in spike latencies in auditory and electroreceptive localizations). Temporal resolutions that support sensory discriminations can range from sub-millisecond timescales, as in electroreception and auditory pitch perception, localization, and echolocation, to much longer timescales that involve the encoding of longer durations associated with event timings (Tucci et al., 2014; Akdogan et al., 2023; Howard, 2024). The patterns can be simple, encoding a one-dimensional attribute, (one or another interspike interval or relative latency) or complex, encoding two or more attribute dimensions, (e.g., a series of intervals, or multiplexed, interleaved intervals, or specific multi-neuron volley patterns).

As with rate-channel codes, temporal codes can be dense or sparse depending on what fraction of neurons are producing temporally-coded patterns of spikes that are related to some specific attribute (Kloppenburg and Nawrot, 2014). Temporal codes can also be dense or sparse in time, depending on what fractions of time contain spikes participating in a particular code. Temporal codes that are sparse in time facilitate multiplexed, interleaving of independent spike patterns. Temporal codes at the levels of neural ensembles and populations can also consist of temporal patterns whose spikes are distributed across multiple neurons.

### 3.2 A taxonomy of neural codes

**FIGURE 1:**
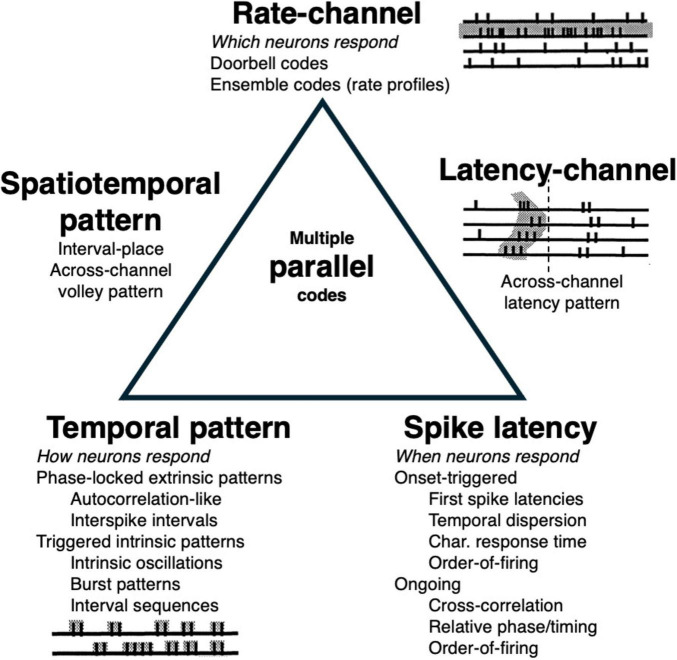
A taxonomy of types of basic temporal codes. Sensory distinctions are conveyed through which neurons respond (channel codes), how they respond with different temporal patterns of spikes (temporal pattern codes) and when they respond (spike latency codes). Combination codes that use two or more types of response attributes lie on the edges. Multiple parallel, semi-independent codes for the same attribute lie in the center.

Taxonomies of different basic types of neural codes have been outlined and discussed in previous papers (Cariani, 1995; Cariani, 1999; Cariani, 2001b; Cariani, 2004). There are many ways to send a message using arrays of channels each of which sends its own train of pulses. A relatively economical taxonomy (Figure 1) divides neural codes into channel codes, temporal pattern codes, and spike latency codes. These are related to three independent aspects of any signal: the channel over which it is sent, its internal structure (e.g., waveform), and its time-of-arrival.

Channel codes are those codes in which the respective identities of the channels are crucial to the meaning of the message. The channels are “labeled lines” such that if the labels are scrambled, so is the message. Temporal pattern codes are those codes in which messages are conveyed through different temporal patternings of spikes rather than through which particular channels are involved, such that they do not require channel-specific labels or connectivities. Spike latency codes are those codes in which the absolute timing of spikes relative to some reference time conveys messages.

The time-of-arrival (latency) of a signal is independent of its internal structure (temporal pattern). Two signals arriving at slightly different times (relative latencies) can provide information concerning the direction and range of objects in various modalities (e.g., in binaural, somatic, olfactory localization; echolocation; and electroreception). Following an onset of a response to an event in one neural population, various other neural populations may respond with characteristic delays, such that recurrent volleys of spikes arriving at a given time after the onset response can indicate that a particular population sensitive to a particular attribute has been activated (e.g., later peaks in event-related-potentials associated with different stages of speech and language processing). The relative latency of the incoming volley can thus indicate the presence or absence of a specific feature.

#### 3.2.1 Types of codes reflect universal neural response properties

This tripartite distinction arises from near-universal properties of neurons as simple integrate-to-threshold elements that are driven by depolarizing input currents. These are:

(1) *monotonically increasing average firing rates* with increased excitatory depolarizing synaptic currents,
(2) *phase-locking*, i.e., spiking preferentially at times when synaptic input currents increase or fluctuate, and
(3) *earlier firing with increased synaptic currents*, i.e., the first spike will be produced earlier with increasing input magnitude

The three properties related to excitation lead, respectively, to rate-channel codes, phase-locked temporal pattern codes, and spike latency codes (Figure 1). At the vertices of the triangle are “pure” codes based on one response property (channel, temporal pattern, timing), whereas at its edges are joint combination codes in which two properties jointly determine a response pattern (which temporal patterns or spike latencies in which channels), and in the center are multiple independent codes (e.g., parallel coding using multiple populations).

**FIGURE 2:**
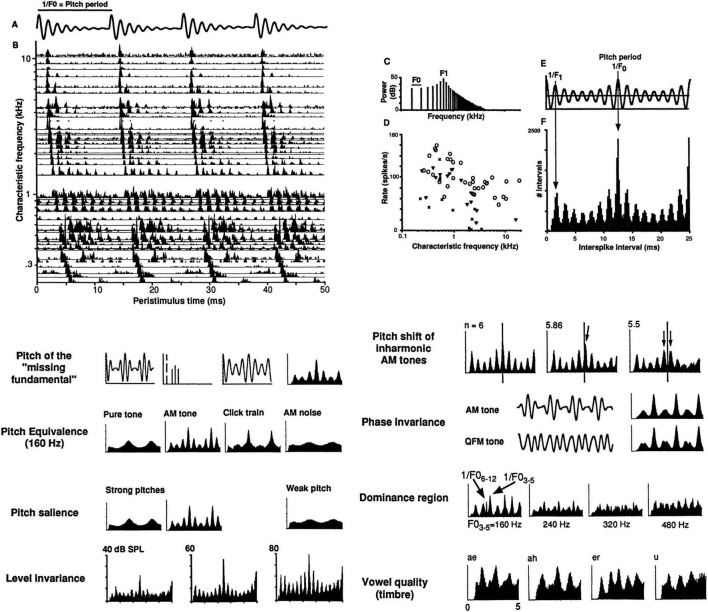
Temporal coding of pitch and timbre in the auditory nerve. Top **(A)** Stimulus waveform: Synthetic single-formant vowel, F0 = 80 Hz, F1 = 640 Hz, 60 dB SPL, 100×, Felis catus, dial anesthesia. **(B)** Post-stimulus time neurogram of 51 auditory nerve fibers (ANFs). **(C)** Stimulus line spectrum. **(D)** Average firing rates vs. characteristic frequencies. **(E)** Stimulus autocorrelation function (ACF). 1/F0 = 12.5 ms = pitch period. 1/F1 = 1.6 ms = period of formant harmonic. **(F)** Population-interval distribution (PID), a.k.a. “summary autocorrelation,” the sum of all-order interval distributions of all 51 individual ANFs, i.e., discarding all cochlear place (CF) information. Arrows indicate peaks related to pitch periods and to the formant frequency. Bottom: Pitch and timbre phenomena predicted by the locations of major peaks in PIDs compiled from spike trains recorded from 50 to 100 ANFs, on the order of 100,000 spikes/plot. First plots: Waveform, line spectrum, and ACF for an AM tone (f_c_ = 640 Hz, f_m_ = 160 Hz) that produces a strong low pitch at its 160 Hz “missing fundamental.” Last plots: PIDs of synthetic two-formant vowels, showing characteristic distributions of short intervals (0–5 ms). See (Cariani, 1999) for additional details.

All three pure types of coding are possible whenever neural inputs are excitatory, so it isn’t surprising when multiple types of candidate codes are found in the same systems (as can be seen in the auditory nerve, Figure 2). It then falls to neuroscientists to determine which codes the system is actually using to realize its various functions.

In addition, many neurons have characteristic recovery times from transient, hyperpolarizing, inhibitory inputs in which the latency of the first spike after a hyperpolarizing pulse is monotonically related to its magnitude (“anode break excitation”). The timing of the second spike can be quite precise. Thus an additional general mechanism that produces spike timing precisions in sensory systems is rebound spike timing from inhibition.

(4) *rebound from inhibition*

If a neuron is driven by an early excitatory wave of inputs closely followed by an inhibitory one, then an onset triggered spike can be followed by a second, precisely-timed anode-break spike to produce a characteristic interspike interval related to the magnitude of the onset transient. This mechanism can support a non-phase locked interval code for the intensity transients: the longer the recovery time from inhibition, the higher the intensity of the transient.

#### 3.2.2 More complex response properties

Of course, many neuronal types can have internal dynamics that are more complex than simple integrate-and-fire neurons, and these can give rise to characteristic spike burst and interburst patterns as well as single spike recovery times. Local networks of neurons, especially if they contain both excitatory and inhibitory elements, as in retinas and olfactory bulbs (Shepherd, 1990), can produce stimulus-triggered characteristic spiking patterns that need not be phase-locked to the stimulus in order to encode different stimulus attributes. Electrical conditioning of single neurons with slow temporal patterns of non-electrical stimulation can cause “assimilation of the rhythm” wherein the neurons produce the conditioned pattern when they fire (Morrell, 1967; Thatcher and John, 1977). Oscillations are temporal spiking patterns that can be produced both by single neurons and local networks (Kopell et al., 2010a). Stimulus-triggered oscillations in characteristic frequency bands can be found throughout the brain (Başar, 1992; Bullock, 1992; Buzsáki, 2006) and potentially play essential functional roles in neural coding (Shamir et al., 2009; Kopell et al., 2010b; Cariani and Baker, 2022; Mignot et al., 2024).

**FIGURE 3:**
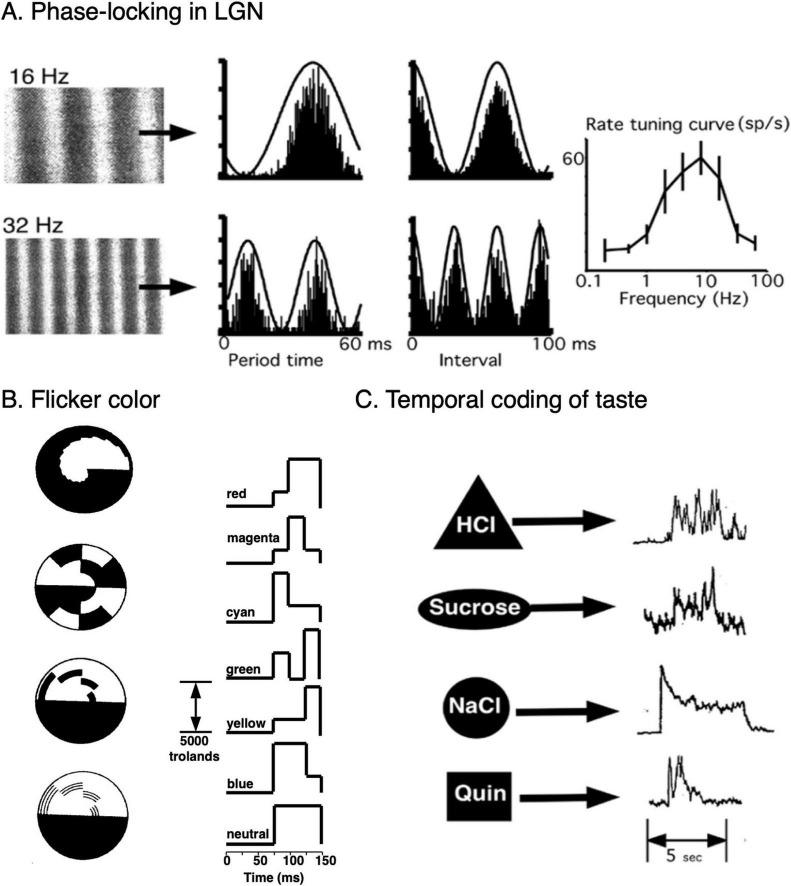
Temporal coding in visual and gustatory systems. **(A)** Responses of a single lateral geniculate neuron of an anesthetized macaque to constant velocity drifting sinusoidal gratings. These correspond to temporal modulations of luminance of 16 and 32 Hz. Aligned period histograms and all-order interspike interval distributions were compiled from 1233 spikes, 5897 intervals (16 Hz) and 495 spikes, 1102 intervals (32 Hz), showing strong phase-locking to luminance modulations. Rate-based spatial frequency (temporal modulation) tuning curve (mean ± s.d.). Estimates of the two temporal modulation frequencies 8 and 16 Hz made by finding the delay associated with the peak in the interval distributions had errors of 0.5 and 2%, respectively. Raw spike train data were provided courtesy of Przybyszewski et al. (2000). **(B)** Monochromatic flicker stimuli that produce subjective colors, the Prevost-Fechner-Benham Effect (Cohen and Gordon, 1949). Left: Rotating disk patterns that were used to study flicker-induced colors top to bottom, by Fechner, Helmholtz, Helmholtz, and Benham. Right. Glow tube monochromatic luminance patterns that produce the corresponding colors (Festinger et al., 1971). Electrical stimulation using the same temporal patterns produce the same colored phosphenes (Young, 1977). **(C)** Typical intrinsic whole-nerve chorda tympani temporal response patterns of different tastant classes in decerebrate rats. The tastants were HCl (0.1M, sour), sucrose (0.5M, sweet), NaCl (0.1M, salty), and quinine (0.1M, bitter). From Covey (1980).

In many cases, electrical stimulation using recorded stimulus-triggered temporal patterns can evoke the corresponding percept. Examples of these types of temporal stimulation and response patterns are illustrated in Figure 3 for gustation (Covey, 1980; Di Lorenzo et al., 2009) and color vision (Festinger et al., 1971; Young, 1977).

### 3.3 Channel-based codes

Temporal codes can be contrasted with channel codes in which different across-neuron patterns of activations convey distinctions. For most of the history of neuroscience, following Helmholtz (Helmholtz, 1885) and Adrian (Adrian, 1928), channel-coding has been the dominant, default assumption for conveying specific informational contents in sensation and perception, cognition, executive functions, emotion, memory, and motoric action. Channel-coded neural representations are described as vectors in which each dimension is associated with a specific neuron (or ensemble or population) and its scalar value with some measure of activation level.

The simplest channel codes are so-called “doorbell codes” in which excitation of highly selective neurons indicates the presence of one particular stimulus, such as a pheromone (one stimulus/neuron). More common candidate channel codes are referred to as “rate-place codes,” in which different levels of activation are signified by different average firing rates, i.e., spike counts averaged over tens to hundreds or more milliseconds, or spiking probabilities, i.e., the probability of a spike occurring within a given temporal window. A firing rate profile of a collection of elements with different selectivities can convey the relative presence of a range of attributes.

A test of whether a given prospective neural code is a channel code is to scramble channel identities/labels and to determine whether this operation changes the functional meaning of the pattern. In spatially-organized maps (e.g., cochleotopic, retinotopic, somatotopic maps) the identity of an individual neuron (which channel, which dimension in the vector) is signified by its spatial “place” in the map). However, spatial organization is not absolutely required, such that neurons spatially dispersed within a population can be organized by their common tunings. Here, “place” can refer to an element’s connectivity position within a neural network (nervous system, brain, region, population, ensemble).

Firing rates are not the only means of indicating channel activations. Other biologically-plausible measures include relative first spike latency (the faster the response, the higher the activation) and relative firing order (earlier responders indicate units with higher activations, subsequent responders indicate lower activations).

### 3.4 Temporal pattern codes

Temporal pattern codes encode distinctions by means of characteristic temporal patterns of spikes, i.e., how neurons respond. In the sensory context, specific temporal patterns are produced by specific stimulus attributes. Temporal patterning can arise in two ways, through phase-locking to a stimulus or through stimulus-triggered response patterns.

#### 3.4.1 Phase-locked temporal pattern coding

What is “phase-locking”? In neuroscience, the term phase-locking is used in several different, though related, contexts. Phase-locking can refer to time-locking of spikes to an external stimulus (stimulus-locking). Unless otherwise noted, this is the sense the term will be used in this paper in discussing sensory coding. Most simply put, phase-locking here means that spike timings are significantly correlated with the waveform of the incoming stimulus. In order to be phase-locked, spikes must occur predominantly during one phase of a stimulating waveform, usually either its positive or negative phase. They can be, but need not be, “entrained” to the stimulus, i.e., one or more spikes produced for each stimulus phase.

Phase-locking can also refer to temporal correlations between neural activations at the levels of individual neurons, ensembles, and whole populations. At the neuron level, these are correlations between spike timings or firing rate fluctuations. At the ensemble level, they may involve measures of multi-unit activity. At the population level, they involve measures of the collective behavior of the population, such as local field potentials and gross potentials. Interneural synchronies at any of these levels can involve spiking and collective events that occur simultaneously (zero-lag) or with some constant temporal offset (lead or lag). Neural-neural phase-locking on population-wide scales can involve temporally correlated brain rhythms and oscillations, be they exogenous (stimulus-locked), endogenous, triggered, or induced.

Phase-locking to motoric actions and their internal representations constitutes yet another application of the general concept. Muscle activations produce corollary discharges as well as muscle and body movements that themselves produce spike patterns in stretch receptors and proprioceptive afferents. These are phase-locked to the movements, especially onsets. These provide both rate and temporal readouts of movements that can be used in active sensing systems to improve tactile, haptic, and visual perception (section “3.9 Temporal coding in active sensing”). In these systems, on the encoding end, spike timing patterns in primary sensory neurons are determined both by the structure of the external stimulus and that of internally-generated movements of sensory surfaces. On the decoding end, neural phase-locked loop (PLL) mechanisms have been proposed to take into account the temporal modulations induced by the movements so as to recover the structure of the external stimulus (Ahissar et al., 2023).

Given the basic response properties of neurons, phase-locking in these abovementioned senses is an almost inevitable, universal process. Whenever depolarizing input synaptic currents from a receptor or another neuron to a neuron fluctuate, neurons fire preferentially when these currents are positive. The spikes are time-locked to the positive phases (positive amplitude portions of the waveform), i.e., they “phase-lock.” In the absence of significant currents, neurons may fire stochastically, e.g., exhibiting Poisson-like “spontaneous activity.” In most receptor systems, receptors produce positive synaptic currents only when cilia are deflected in one direction, such that alternating motions (e.g., vibrations, aperiodic fluctuations) produce half-wave rectified voltage and current waveforms. As a consequence, spike timings are correlated with the stimulus waveform as it has been filtered through its passage through sensory organs and receptors.

If sensory neurons phase-lock to the stimuli that excite them, then the time structure of the stimulus waveform is impressed on that of the produced spike trains. This in turn means that any attributes closely associated with the time structure of the stimulus waveform will have correlates in the resulting patterns of spikes. This includes auditory and visual event onsets and durations, event rhythmic patterns (musical rhythm, speech prosodic rhythms), periodicity (auditory pitch, flutter-vibration, visual flicker frequency), spectrum (auditory vowel quality, spectral tilt/timbral brightness), onset dynamics (musical attack), and modulation spectrum (consonantal distinctions, infrapitch). If a feature is in the waveform, and within the frequency range of phase-locking, it will have a phase-locked temporal pattern correlate in response spike trains. More succinctly, “if it’s in the waveform, it’s in the spikes” somewhere, provided that there is at least some phase-locking in the system.

Phase-locking effectively encodes the time structure of periodic, near-periodic, and aperiodic stimuli, and does not depend on any particular waveform type (sinusoid, impulse, square wave, noise), but it is limited in the frequencies it can track by the low-pass filtering imposed by sensory organs and receptors and jitter introduced by synaptic transmission. In mammalian cochleae, filtering successively reduces the AC components of high frequency pure tones, rolling off at a few thousand Hz, with synaptic jitters on the order of 100 microseconds. The two factors reduce usable phase-locking of mammalian auditory primary neurons to somewhere between ∼3–8 kHz, depending on species (there is debate about the upper limit in humans). In barn owls, phase-locking exists up to ∼10 kHz (Koppl, 1997). Weakly electric fish have special adaptations (electroreceptors, gap junction synapses, coincidence detectors with many input lines) that reduce jitter, enabling them to detect and produce electric signals with sub-microsecond precisions (Carr et al., 1986; Heiligenberg, 1991; Carr, 1993b; Heiligenberg, 1994).

Phase-locking is found in nearly every sensory system, with spiking patterns of many sensory neurons mainly reflecting the fine structure of the impinging stimulus itself (e.g., vibrations). In active sensing systems there is also phase-locking to movements of the animal and its sensory surfaces, such that spiking patterns also reflect the temporal structure of those movements [e.g., gross and fine eye movements (Rucci et al., 2018), whisker movements, sniffing cycles, bodily accelerations]. Temporal spiking patterns related to the timings of movements can be used to separate out those patterns that reflect properties of external objects from those that reflect movements (see section “3.9 Temporal coding in active sensing”).

In everyday life, very few stimuli, if any, are completely static. In the case of vision, the eyes are in intermittent saccadic motions and constant micro-motions such that the retina is constantly producing short-time representations of images in the form of spatial patterns of phase-locked spikes. When images are stabilized on the retina, presumably phase-locking is abolished such that neurons revert to Poisson-like spiking, and form perception rapidly disappears (Ditchburn and Ginsborg, 1952; Coppola and Purves, 1996). This is highly suggestive that visual form perception critically depends on phase-locking. See also discussion in section “9.1 Form vision.”

Phase-locked time structure is largely preserved when auditory waveforms and visual images are infinitely-peak-clipped, i.e., reducing waveforms and images to binary amplitudes (below average values are set to 0, above-average values to 1). Remarkably, speech intelligibility of infinite-peak-clipped sounds is little affected (Licklider, 1951b) and images remain quite recognizable. Patterns of acoustic discontinuities and visual contrasts (edges), not graded amplitudes, are most important for perception of auditory qualities and visual form. Phase-locking is arguably the simplest and most effective means of encoding patterns of temporal and spatial edges.

Interspike interval codes are the simplest temporal pattern codes. First-order interval codes consist of distributions of time intervals between consecutive spikes, whereas all-order interval codes instead count intervals between consecutive and non-consecutive spikes. All-order distributions are equivalent to the autocorrelation functions of spike trains. Interval sequences, such as triplet codes consisting of sequences of two intervals (Lestienne, 1996; Lestienne and Tuckwell, 1998), can be described in terms of triple autocorrelations (Yellott and Iverson, 1992). Longer interval sequences can be described in terms of still higher order autocorrelations (Victor and Conte, 1996).

Phase-locked interval codes can also indicate stimulus intensity in the degree to which the activity of a population exhibits a common time pattern. In the auditory nerve, the proportion of spikes that are phase-locked vs. those “spontaneous” spikes that occur at random phases increases monotonically with sound level and its perceptual correlate, loudness (one expects an analogous situation for contrast and phase-locking in retinal elements). As a consequence, peak-to-mean ratios in population-interval autocorrelation distributions at the level of the auditory nerve are indicators of ratios of driven, correlated, phase-locked responses to uncorrelated, spontaneous activity (Figure 2).

#### 3.4.2 Stimulus-triggered temporal pattern coding

In addition to phase-locked temporal response patterns, specific stimuli can trigger different endogenous response patterns in neurons. These characteristic responses need not have any necessary correlation with the stimulus time structure beyond the universal event-attributes of event onset, offset, and duration. Examples include temporal coding of taste and color (Figure 3) that are further discussed in sections “9 Vision” and “10 Chemical senses: gustation and olfaction.” Characteristic response patterns can also come in the form of bursting patterns (burst length and/or interburst intervals) and intrinsic oscillations of different frequencies or combinations of frequencies.

#### 3.4.3 Spike correlation codes

Whereas the simplest temporal patterns can be interspike intervals produced through phase-locking to periodic stimuli or produced by triggered oscillations, more complex, specific patterns of spikes in the form of spike volley codes or spike correlation codes are also possible, e.g., (Fetz, 1997). Such correlation codes would also encompass volley patterns consisting of sets of characteristic spike latencies, making them varieties of spike latency coding. If the volley patterns are independent of channel identities, then they are purely temporal codes. If channel identities matter, then they are combination channel-temporal pattern or channel-latency codes in the taxonomy.

### 3.5 Spike latency codes

Spike latency codes rely on specific timings of spikes relative to some internal reference time. First-spike latency codes can encode the intensity of a stimulus onset or amplitude transient with high fraction-of-a-millisecond precisions (Phillips, 1993; Heil, 1997; Heil, 2004), which can be compared with coarser estimates using information-theoretic counting window duration methods (Kayser et al., 2010). Remarkably, first spike jitters for onsets at auditory cortical stations can be comparable to those in primary auditory neurons, due presumably to repeated processing by neurons performing coincidence detection operations on many independent inputs – jitter is preserved or reduced by virtue of the Central Limit Theorem. Shorter response latencies of individual neurons and narrower temporal dispersions of spikes indicate higher change of amplitude (contrast). Here the internal reference time may be the timing of some threshold of onset-correlated spiking across a population. In the retina, first spike latencies can encode contrast, i.e., edges and contours (Gollisch and Meister, 2008; Gutig et al., 2013). Ensemble- and population-wide oscillations can also serve as reference times, with oscillatory phase offsets (time delays, relative latencies) encoding different stimulus attributes in olfaction and other modalities (Hopfield, 1995; Masquelier et al., 2009). Such phase-offsets are widely observed in the hippocampal coding of different locations (places) in maze navigation tasks (Skaggs et al., 1996).

Different subpopulations of neurons can have different characteristic response times, such that peaks with different latencies in population-wide responses can indicate the degrees to which different subpopulations are activated. Some early theories of color, noting the different latencies of peaks in averaged evoked responses to different wavelengths of light, postulated mechanisms of this sort.

Joint channel-temporal codes combine temporal patterns and/or latencies with labeled lines. For example, in the auditory nerve the appearance of interspike intervals associated with a common frequency component across many channels can encode the relative amplitude of that component relative to others. A particular set of interspike intervals or burst patterns appearing in some specific characteristic frequency channel, such as those associated with some modulation frequency or event-onset rhythms, can constitute an auditory interval-place representation (Voigt et al., 1982). Similarly, relative spike latency can replace firing rate as an indicator of which channels are most highly activated. Here the channel(s) with the shortest response latencies serve as the best estimates of attribute values. Because average firing rates and shortest spike latencies often occur together, the two codes can be difficult to disambiguate as to which are causal to function.

In phase-locked sensory systems, various localization functions can be achieved by comparing the relative arrival times of similar waveforms at different body (receptor) surfaces, i.e., different channels. This strategy is seen for localization attributes in many different modalities: electroreception, audition, somatic, olfaction, and gustation. In binocular vision, interocular delays are interpreted as depth cues, as the Pulfrich Effect illusion suggests (Graham, 1966; Carr, 1993b). Perhaps the most widely appreciated example is binaural localization in the horizontal plane. For example, in human binaural localization different directions of sound sources in the horizontal plane produce differences of sound arrival times between the two ears that range up to roughly 500 microseconds. Due to phase-locking, these time differences are faithfully preserved in spike trains of the two auditory nerves, and through highly secure synapses in those of spike trains produced by neurons in the anterior cochlear nucleus. In effect, a neural temporal cross-correlation function is computed using delay lines and binaural coincidence neurons in the auditory brainstem (Jeffress, 1948; Cariani, 2011). The interaural delay channel in the binaural array that has the highest number of coincidences, i.e., the highest firing rate, indicates the interaural delay and hence the direction in the horizontal plane.

### 3.6 Firing sequence codes

Related to spike latency codes are firing sequence codes in which the temporal order of responses in different channels encodes some stimulus attribute. This is a joint channel-time code by virtue of the necessity of labeling the channels, but one in which the temporal dimension has ordinal values rather than metrical ones Such codes have been proposed for vision in light of experiments that strongly suggested short neural processing windows for form vision (Thorpe, 1990; Coppola and Purves, 1996).

A primary advantage of such codes is that they only require one spike per channel, permitting rapid coding of images within very short time windows (Van Rullen and Thorpe, 2001; Van Rullen et al., 2005). The one-spike-per-neuron feature ameliorates the many problems posed by the movement of the image relative to the retina for computing spike rates over longer time windows of 50 ms or more. To avoid confusion, note that the “interspike interval” (“ISI”) code in Van Rullen and Thorpe (2001) is a *mean* interspike interval measure, i.e., an alternative way of computing spike rate, rather than a temporal code, as we use the concept here. Such firing sequence codes are also relatively insensitive to image degradations (Delorme and Thorpe, 2001).

Order of firing codes also potentially solve what is known as the Hyperacuity Problem (Thorpe et al., 2004; Altes, 1989). Many rate-place codes are plagued by this problem, where the acuity of perceptual systems, as evidenced in controlled psychophysical experiments and behavioral observations, is over an order of magnitude finer than the rate receptive fields of the most selective neurons in the corresponding primary sensory neurons (Rieke et al., 1997).

However, it is also often overlooked that phase-locked temporal codes (pattern or latency) often have precisions that are orders of magnitude better than their firing rate counterparts that lead to predictions of much smaller Weber fractions, e.g., in the auditory nerve (Siebert, 1968; Heinz et al., 2001). Once spike timing information enters the picture, this “hyperacuity problem” goes away. Temporal coding also can solve analogous hyperacuity problems in other systems, such as in visual representations of space (Rucci et al., 2018), vernier actuity (Assa et al., 2025), and haptic localization of objects using vibrissal systems (Knutsen et al., 2006) Other mixed-time-place codes that use spatial patterns of synchronous, phase-locked, temporally correlated spikes could also operate on one-spike-per-channel constraints [cf. spike correlation codes of Jacobs et al. (2009) and some temporal encoding assumptions in Ahissar and Arieli (2012)].

On the belief that such synchronies are redundant with respect to retinal rate-place profiles and sub-optimally reduce rate-contrasts across neighboring receptive fields, some workers eliminated synchronized spikes from consideration (“decorrelation”) in attempts to improve rate-place codes (Kuang et al., 2012). If one did this in the auditory nerve (Figure 2), there would be almost nothing left.

Firing sequence codes are perhaps best conceptualized in terms of synfire chains (Abeles, 2009) in which spike timings and interneural delays cause chains of coincidence elements to fire. Different firing orders activate different sets of synfire chains. But it is difficult to imagine how visual images or auditory scenes or olfactory mixtures could be robustly constructed from ensembles of firing sequences. How well do these representations fare in terms of perceptual invariances (e.g., similar triangles of different colors, contrasts, positions, sizes)? How do they fare in terms of common percepts in the face of other changing attributes? For example, F0-pitches of musical notes are highly invariant with respect to sound level, direction, attack, instrument timbre – one can play the same note a bit louder, move around, play staccato or legato notes, or even change instruments and the pitch remains the same. But, because neurons are typically weakly sensitive to multiple parameters, these manipulations will certainly change many firing orders of neurons in the auditory pathway.

The particular synfire chains that are activated by particular stimulus conditions are intimately tied to particular channels and transmission paths. Consequently, they encounter many of the difficulties of rate-place and feature-detector-based representations. Sequence codes have the merit of being invariant under some time scale transformations, but the number of alternative chains is quite high, making the dimensionality of representations based on them many orders of magnitude higher than the structure of their corresponding percepts and perceptual scenes.

Dynamical system trajectories can be regarded in terms of firing times and sequences within whole neural populations. Their phase spaces can consist of time-channel matrix of spike timings that may have many of the same aforementioned difficulties as simpler firing sequences. For the sake of parsimony, some principle needs to produce natural equivalence classes amongst the astronomical number of trajectories observed within the phase space that map easily to mental states and behaviors.

To our knowledge, no completely comprehensive theory of the full space of possible spike codes has yet been proposed that encompasses all of the codes discussed above as well as still others yet to be proposed. We believe that such a synthesis is possible and would be useful. Perhaps (Victor and Purpura, 1996) comes closest. Such a systematic space would need to cover metrical and non-metrical pattern codes, ordinal codes, combination codes, sequence codes, as well as moments of distributions of coded variables (e.g., means, variances, skewnesses, kurtoses of interval or firing rate distributions) and trajectories through dynamical systems phase spaces (Mazor and Laurent, 2005). This is not to mention high dimensional, often inscrutable, codes derived from deep learning and convolutional neural networks (Mathis et al., 2024). The expectation is that biological systems have found much more elegant, simpler solutions.

### 3.7 Coding transformations and parallel codes

Temporal and channel codes are not mutually exclusive (Masuda and Aihara, 2007). They can exist at different stages of sensory pathways, with coding transformations occurring from peripheral to central stations. The transformations can convert temporal patterns to rate-place channel patterns, as in time-delay neural networks. Central neural phase-locked loops were originally proposed as mechanisms for converting spike latency-channel representations to rate-channel representations (Ahissar, 1998; Ahissar et al., 2000). Rate-place patterns can also be transformed to temporal patterns, as in spike latency volley pattern codes, mixed latency-place codes, order-of-firing channel codes, and central pattern generators. Evidence for rate-place coding at one stage in sensory pathways does not rule out its conversion to some sort of temporal code, e.g., spike latency or firing order, at higher levels.

Temporal and channel codes can also exist as parallel, partially redundant coding systems. For example, in thermoreception, different neural subpopulations are thought to respond to hot and cold stimuli [section “11 Cutaneous sensations (pain, itch, temperature)”]. High acuities may be achieved using temporally-coded information and active-sensing central mechanisms, whereas lower acuities may be found when sensory systems are forced to rely solely on less precise spatial, channel-based codes (Gamzu and Ahissar, 2001; Ahissar and Arieli, 2012).

Often first spike latencies and firing rates are highly correlated making it difficult to disambiguate which aspect of response spike trains is causal to function. Finally, there can be joint, combination temporal and spatial, channel profile codes (Figure 1) in which temporal patterns or relative spike latencies/firing orders mark which channels are most activated. Here temporal markers play the role of firing rates in differentiating channel activation patterns. Two examples are a putative interval-channel code in the auditory system (Voigt et al., 1982) and latency-place codes for somatic localizations (von Békésy, 1967).

### 3.8 Multiplexing of temporally-coded signals in the same channels

Contrary to what might have been expected 75 years ago, most neurons in the brain have turned out to be not highly selective, unitary feature detectors. The dogma of one feature-one neuron has gradually eroded away, except in very specialized sensory domains. Many cortical neurons respond to stimuli in multiple modalities [e.g., visual and tactile information (Bieler et al., 2017)]. Even cortical neurons within a single modality respond to changes in multiple perceptual attributes within that modality, e.g., (Bizley and Walker, 2010). It is hard to see how individual features get sorted out at the cortical level. Neurons in supramodal areas often may respond to multiple objects having different sets of multi-modal perceptual, cognitive, emotional, motivational, and action-related attributes. The ubiquity of multi-valent “mixed selectivity” neurons poses deep problems for theoretical neuroscience (Fusi et al., 2016). It is possible that complex, central, multiplexed temporal codes could solve some of these problems (Cariani and Baker, 2022; Baker and Cariani, 2025).

A prime advantage of codes that do not require labeled lines and consequently, highly specific connectivities and transmission paths is that these coding schemes “liberate the signals from the wires”, thereby enabling broadcast and selective reception strategies for information integration (Cariani and Baker, 2022; Baker and Cariani, 2025). Another advantage of temporal codes is that they lend themselves to multiplexing of signals, i.e., multiple types of pulse coded information can be sent along the same axonal lines at the same time. Multiplexing also relaxes the strict transmission path constraints that channel codes appear to require. One spike train can thereby convey “multiple meanings” (Chung et al., 1970; Chung et al., 1974; Wasserman, 1992; Cariani, 2004).

In sensory systems the different signals can be related to different independent attributes of a single object, event, situation or body location. An example would be multiplexed coding of the various cutaneous senses, such as vibration, pain, itch, temperature, and pressure (Emmers, 1981; Woo et al., 2022). On the motor side, different multiplexed signals enable a single neuron to independently control different muscles (Bittner, 1968). Along these lines, differential filtering of temporal patterns of action potentials at axonal branchpoints could provide an intraneural mechanism for demultiplexing spike trains into independent components (Raymond and Lettvin, 1978; Pratt, 1990).

Multiplexing in the time domain also potentially simplifies problems of scene analysis (perceptual organization, segmentation and binding of attributes of multiple objects). Time-division, frequency-division, code-division, and oscillatory phase multiplexing of spike train signals are different strategies for solving these problems. More complex, multiplexed, multimodality, and multiscale central codes, including temporally-organized “packets” (Luczak et al., 2015) are also possible. We have discussed many of these alternatives in greater depth elsewhere (Cariani, 2004; Cariani and Baker, 2022; Baker and Cariani, 2025).

Many temporal codes are relatively sparse in time and therefore only minimally interfere with one another. Some temporal codes permit interleaving of spike patterns, whereas for other interval codes interleaved spikes may alter the encoded meanings of the pulse train signals. For example, all-order interval codes are impervious to added or subtracted spikes. These codes consist of time intervals between pairs of both consecutive and non-consecutive spikes. Distributions of all-order intervals are equivalent to autocorrelations of spike trains (section “6.1 Basic auditory qualities”). The neural code for pitch at the level of the auditory nerve appears to be based on such autocorrelation-like interval codes (section “6 Audition,” Figure 2). In contrast, adding or subtracting spikes for first-order interspike interval codes, i.e., intervals between only consecutive spikes, will change the encodings and therefore the functional meanings of the spike train messages.

Multiplexing in the time domain also potentially simplifies problems of scene analysis (perceptual organization, segmentation and binding of attributes of multiple objects). Arguably some means of multiplexing signals is needed for integrating (binding) many different types of information in cognitive representations and memory traces of objects, events, situations, and internal procedures. When multiple objects drive overlapping channels (frequency channels in audition, spatial channels in vision) temporal correlation structure can be used to separate them (Cariani, 2004).

### 3.9 Temporal coding in active sensing

Active sensing involves use of bodily actions to reveal to the senses the structure of objects and events in the environment. A simple example is to sense the properties of an irregular or smooth surface by running one’s finger over it. Here both stimulus structure (surface characteristics) and movements of sensory surfaces (what speed and pressure are used) determine temporal spiking patterns in primary mechanoceptive neurons.

General theories of perception can be classified according by their assumptions regarding the relation of incoming sensory information to internal (cognitive, motivational, mnemonic, and motoric) states (see (Ahissar et al., 2015) for a taxonomy of theories from the active sensing perspective). Sensory systems that mainly rely on bottom-up, environmentally determined information flows from sensory surfaces (e.g., auditory systems) are labeled “passive mechanisms”, whereas those that function using both bottom-up flows and deliberate, self-generated actions (e.g., vibrissal systems) are labeled “active mechanisms.” Modalities in which active sensing plays critical roles can be seen as “closed-loop convergence processes” (Saig et al., 2012; Ahissar and Assa, 2016) in which “action-dependent perceptual invariances” can be achieved.

In this paper, our sense of “sensory coding” concerns what information is available in peripheral spike activity patterns, irrespective of whether they are produced by external stimuli (objects, events) or self-generated motions. Both passive and active sensing modalities depend on temporal coding in the form of spatial and temporal patterns of spike timings.

In order to gain information about the surface, neural response patterns related to the surface properties and not to the bodily movement need to be separated. Central neural mechanisms that demodulate (“decode”) the stimulus-movement mixture are therefore needed. Feedback mechanisms in the form of neural phase-locked loops have been proposed by Ahissar and co-workers to carry out this role of taking into account the contributions of bodily movements so as to recover stimulus attributes (Ahissar et al., 2023).

Early on characteristic intrinsic low-frequency oscillatory activity was discovered by Ahissar and Vaadia (1990) in the somatosensory cortex of awake monkeys. These findings subsequently led to models of active sensing in rat whisker systems based on central oscillators and thalamic phase comparators. These “compare cortical timing expectations with the actual input timing and represent the difference by their population output rate” (Ahissar et al., 1997).

By this proposed mechanism, different spike latencies relative to the timing of movements in sensory peripheries can be converted to central firing rate codes (Ahissar, 1998). Here different whisking phases correspond to spike timing differences, whereas whisking frequencies correspond to time lags and interspike intervals (instantaneous frequencies). Active coding models successfully predict performance declines when whisking frequencies fall outside the working frequency ranges of central oscillators that support phase-locked loop mechanisms and latency-to-rate conversions (Knutsen et al., 2006).

In taking account of the state of sensory organs, central mechanisms can support perceptual invariances with respect to actions, such as self-motions (Wallach et al., 2016) and sniffing dynamics (Jordan et al., 2018). In these mechanisms, internal induced oscillations can also support prediction and anticipation of expected inputs on upcoming cycles with subsequent computation of expectancy violations that can indicate changes in the external world.

## 4 What is encoded?

Different sensory modalities convey different types of distinctions regarding the sensed-state of the body (intero-reception) and the world external to it (extero-reception). Intero-receptive modalities convey information related to the bodily attributes of pain, irritation (itch), internal temperature, digestive, circulatory, respiratory and immunological states, and muscle fatigue, as well as body positions (proprioception, stretch and position receptors), self-motion and orientation (vestibular). Extero-receptive modalities in humans include auditory, visual, tactile, vestibular, thermal, olfactory and gustatory distinctions related respectively to patterns of external sounds, light, skin contacts and vibrations, imposed motions, external temperature, inhaled and ingested chemicals. Other animals also have additional extero-receptive sense modalities such as magnetoreception and electroreception.

Perception has strong modal structure and within it a dimensional structure of different attributes (Boring, 1942). Many of the attributes of each modality have parallels with those of other modalities, such that these parallels can be grouped into a few categories:

1. *basic sensory qualities*, such as auditory pitch and timbre, visual texture and color, taste, smell, sharp vs. dull tactile sensations, salty vs. bitter vs sour taste, hot vs. cold, pain, and itch, – “what sensations”?
2. *intensity*, such as loudness, pitch and timbral salience, lightness (brightness), contrast, color saturation, as well as tactile, thermal, and nocioceptive intensities – “how much”?
3. *spatial forms*, such as 2- and 3-D visual and tactile shapes – “what shape, what object”?
4. *temporal forms*, such as auditory rhythmic patterns, and temporal sequences (e.g., bird calls, speech streams, sequences of changing qualities of all sorts) – “what temporal pattern of events”?
5. *directions of stimuli relative to body surfaces*, such as apparent directions of external objects and sources in visual, auditory, olfactory, and electroreceptive spaces and apparent location of stimuli contacting one’s body – “where”?
6. *apparent distance (range)* of external objects and sources in visual, auditory, olfactory, and electroreceptive spaces, such as is determined by visual parallax for depth perception and auditory echolocation, as well as from prior knowledge of object sizes and intensities as a function of distance – “how far”?
7. *apparent sizes of external objects* – “how big?”
8. *apparent motions of objects and sources* – “how fast? where are things headed?”

The dimensional structure coupled with common kinds of attributes compels consideration of a neuro-phenomenological isomorphism hypothesis vis-à-vis neural coding. Here each modality has its own correlation structure of incoming sensory fluxes impinging on primary sensory surfaces, as well as its own receptor types, proximal circuits, afferent and efferent pathways, and thalamocortical organizations, albeit with many commonalities across modalities.

A working hypothesis is that every independent perceptual quality within each modality has a corresponding, independent dimension of neural coding. The modal and dimensional structure of perception falls out of 1) which aspects of the external world to which receptors are sensitive, 2) the spatiotemporal correlation structure of the incoming sensory fluxes on receptor surfaces, 3) the nature of the neural codes that convey these correlation patterns more centrally. The modal structure of perception arises from differences in the patterns the sensory receptors encode as well as the neural codes and processing operations that are required for information given via that modality to guide effective action. The dimensional structure of perception mirrors that of the correlation structures and neural codes in each modality.

Where there are similarities between correlation structures and neural codes, there will exist inter-modal parallels (e.g., similar cross-correlation mechanisms for visual, auditory, and tactile localizations (von Békésy, 1964b,^1967^). In active sensing systems, such as touch and vision, cross-modality similarities may also arise from common central mechanisms for separating sensory contributions of self-movements from those related to properties of external objects (Ahissar et al., 2023).

## 5 Coding in specific sensory systems

In almost every sensory system there is some evidence for the role of temporal discharge patterns for conveying complex stimulus qualities in a host of human and animal modalities: audition, vision, the vestibular sense, olfaction, gustation, the cutaneous senses of mechanoreception (vibration, pressure), nocioception (pain), thermoreception, proprioception (body position and movement, muscle position and stretch receptors, haptic perception), electroreception, visceral sensations, magnetoreception, baroreception, and perhaps yet others waiting to be discovered.

Often there is evidence for both temporal and rate-channel coding. In such cases, the two can be difficult to disambiguate, i.e., to determine which type of information is causally related to function. In the past, often, investigators stopped looking for possible alternative, temporal codes once evidence for rate coding and neuronal specificity were found. In part, this occurred because, historically, concepts of rate codes and their subsequent interpretations by the rest of the brain have been more easily and universally understood, than those involving temporal codes.

## 6 Audition

Audition is a general-purpose perceptual system used by humans and animals to detect, discriminate, and recognize sounds and to localize their sources in the external environment so as to guide behavior. Its major functions involve hunting prey, avoiding predators, navigation, intraspecies communications (speech, animal vocalizations), and, in humans, self-modulation of psychological states (music).

Audition is a phase-locked sensory system par excellence. It is a sense modality whose psychophysics and neurophysiology, including temporal coding, have been intensively investigated. Its literature is voluminous. Good places to start are (Schnupp et al., 2011; Moore, 2013). [Moore, 2013, reference?]

Ubiquitous, abundant, and usable phase-locked temporal spike patterns can be found at the first stage of neural representation, in the auditory nerve (Figure 2B). In humans usable spike timing information is thought to existwhere they exist for periodicities roughly up. Phase-locking rolls off at high frequencies, its upper limit varying with species. In humans auditory percepts such as octave matching and the existence region of musical tonality (Cariani, 2019) suggest an upper limit of 4–8 kHz, depending on the individual listener.

However, but like many other sensory systems, as one proceeds up the pathway, spikes are more and more temporally sparse, jitters accumulate, and spikes related to multiple kinds of information intervene. Consequently phase-locking becomes progressively less evident at cortical stations, where nevertheless phase-locking can still be observed up to a few hundred Hz (Cariani, 1999; Cariani and Micheyl, 2012), sufficient to cover human voice pitches, low-frequency envelope periodicities, and event onset timings in rhythms.

Temporal codes can be fine or coarse depending on the precision of spike timings. They can depend on timings of individual spikes, potentially yielding submillisecond precisions, or on temporal patterns of spike rates or spiking probabilities within populations, with precisions in tens of milliseconds or more.

Whenever sounds are presented to the ear at moderate to high sound levels, large swaths of the auditory nerve phase-lock to periodicities in the acoustic stimuli, including fundamental frequencies, individual frequency components, low frequency envelopes of interacting components, as well as aperiodic temporal patterns of acoustic transients and event-onsets. Analogous phase-locked responses, albeit with differences of frequency range and phase-locking precisions, can be found in primary auditory neurons in mammals, birds, reptiles, amphibians, fish, and insects. Spike timing plays a major role in the coding of basic auditory attributes involved in detecting, localizing, and recognizing sounds in the external world, with humans reliably receiving speech, and listening to music.

### 6.1 Basic auditory qualities

A substantial body of evidence exists for temporal coding of basic auditory qualities of music, such as pitch, consonance/roughness, timbre, note duration, and rhythm, and those of speech, such as voice pitch, vowel and consonantal distinctions, and speech rhythms.

*Pitch.* Pitch is a primary auditory attribute related to the dominant repetition period (periodicity) of a periodic sound. This is variously known as the “low” pitch at the fundamental F0, F0-pitch, virtual pitch, and musical pitch (Cariani, 2019). It has a long history and a rich set of precisely measured perceptual phenomena that enable various neural coding hypotheses to be tested.

Both sinusoidal, pure tones and harmonic complexes of human voices and tonal musical instruments produce strong pitches. The strongest, most comprehensive, physiologically-grounded models for both pure (Goldstein and Srulovicz, 1977; Heinz et al., 2001) and complex tone pitches (Meddis et al., 1990; Meddis and Hewitt, 1991; Cariani and Delgutte, 1996; Cariani, 1999) are those that use phase-locked spike timing information in the form of auditory nerve fiber (ANF) interspike interval distributions (Figure 2). The latter models estimate the pitches that will be heard from the most numerous all-order interspike intervals present in the auditory nerve. This is a phase-locked temporal pattern code. Due to phase-locking the time structure of the neural spike trains are highly correlated with that of the stimulus. For physiologically-resolved harmonics, spikes are phase-locked to individual harmonics (“temporal fine structure”), whereas for pairs and groups of unresolved harmonics, spikes also phase-lock to the lower-frequency envelopes (“modulations”) created by beating harmonics. Interspike intervals created by phase-locking to individual harmonics and to envelopes permit representations of pitch for resolved and unresolved harmonics.

Because the all-order interval distributions are autocorrelations of these spike trains, when they are added together, their population-interval distribution (PID, 2F) resembles the stimulus autocorrelation function (ACF, 2E), which carries the same information as the stimulus power spectrum.

The population-wide interval distribution (PID) is therefore a purely temporal code that does not depend on which characteristic frequency channels produced which intervals, and as a general-purpose neural autocorrelation-like representation, it conveys information about both stimulus periodicity and spectrum.

As a result, the peaks in the neural PID indicate all the periodicities in the stimulus waveform, sufficient to precisely predict the dominant periodicity (the fundamental period 1/F0 and its multiples, here 1/80 Hz = 12.5 ms) as well as other periodicities (the period of the harmonic at the formant 1/640 Hz = 1.6 ms). The precisions of estimates derived from a few thousand ANF spike times produces F0-pitch estimates that have errors on the order of half a percent in frequency, in the same ballpark as human listeners.

These models also predict a host of other pitch phenomena, such as pitches of missing fundamentals, pitch equivalences between stimuli with different amplitude and phase spectra, pitch invariance with respect to level, pitch shifts of inharmonic complex tones, the dominance region for pitch, and spectral edge pitches (not shown). The only pitch phenomena that are clearly not predicted are subtle F0-pitch shifts of harmonic complexes with one slightly mistuned individual harmonic (Dahlbom and Braasch, 2020) and not-so-subtle Zwicker pitches (Gockel and Carlyon, 2016), which are auditory afterimages that probably have a central rather than peripheral origin.

*Spectrum.* Because the PID also temporally encodes spectral information below ∼5 kHz, it also can serve as a representation for vowel timbral space (Palmer, 1990). In so-called “double vowel” experiments (Cariani and Delgutte, 1993), we found that we could accurately identify single and double vowels by comparing the correlations of neural PIDs with stimulus autocorrelations of single-vowels (Cariani and Delgutte, 1993). The single-vowel PID profiles are shown in the last plots of Figure 2 (Cariani, 1995).

Temporal coding also may explain how pitch and timbral commonalities (e.g., different instruments playing the same note, the same instrument playing different notes) can be extracted. By simply by multiplying the summary autocorrelations of each of two conditions, their common peaks associated with either pitch or spectrum reinforce each other (Cariani, 2001a). Time domain representations and correlation-like processing operations can simply auditory scene analysis, a.k.a. Gestaltist object formation, segmentation and binding, Cherry’s “cocktail party problem”, using the different voice pitches of speakers to separate out the different voices (Cariani, 2004). Using correlations between neural PIDs and stimulus autocorrelations and neural timing nets, the single vowel constituents of concurrent double vowels with the same and different voice F0-pitches can be accurately identified with patterns that resemble human performance (Cariani, 2001c).

*Loudness.* Because the fraction of driven, correlated spiking across the whole auditory nerve (vs. uncorrelated, spontaneous activity) increases monotonically with sound level, degree of spike correlation within a population is a potential neural correlate of stimulus intensity (loudness). Here correlation index could be a useful metric for testing this conjecture (Joris et al., 2006).

*Musical tonality and rhythm.* Other qualities important in tonal music, such as octave similarity, roughness, consonance (harmonicity, tonal fusion), musical interval ratios, and tonal hierarchies may be explained in terms of autocorrelation-like temporal codes based on interspike intervals (Cariani, 2019). Here the fraction of intervals related to overlapping subharmonics determine perceptual distances which form a tonality space (see also (Leman and Carreras, 1997)). Although melodies are entirely recognizable when transposed (multiplying all note F0-frequencies by a constant ratio), this recognition breaks down for melodies consisting of notes above ∼4 kHz, near the uppermost note on the piano. For most listeners, octave equivalence also breaks down at this frequency, which may be the upper limit of usable phase-locking for within-CF-channel interval codes in most humans (a few of whom can make octave equivalences at somewhat higher frequencies).

*Speech.* Basic phonetic distinctions depend on quasi-stationary spectral shapes in the case of vowels and transient amplitude and frequency patterns in the case of consonants. Vowel formant space is well represented in autocorrelation functions and neural population-interval distributions of the auditory nerve. Neural correlates of vowel category boundaries of specific languages are presumably located at higher levels of the auditory pathway. The neural coding of consonantal distinctions, on the other hand, are much less well understood because they involve spike patterns related to acoustic contrasts and discontinuities (onsets, offsets). This makes them amenable to latency coding relative to population onset responses.

Speech is highly redundant, with many different kinds of cues and representations, such that limited recognitions can be achieved with reduced sets of these. Adequate, but suboptimal levels of speech intelligibility can be achieved using information in the modulation spectrum (Fogerty et al., 2023), i.e., low-frequency (<50 Hz) envelope fluctuations of higher frequency carriers (Ghitza et al., 2012). Temporal patterns related to slower modulations are mostly the information that cochlear implant users must rely on for consonant identification and speech intelligibility. Although the modulation spectrum appears sufficient for minimal recognition of consonants (Chait et al., 2015; Teng et al., 2021), reduced-channel vocoder experiments (Shamma and Lorenzi, 2013) suggest that although temporal fine structure cues are more resistant to noise, information related to either temporal fine structure or modulation envelopes can be used.

Neurons coarsely-rate-tuned to low frequency modulations of higher frequency carriers are found throughout the auditory pathway (Schreiner and Langner, 1988; Langner, 1992), but a rule of thumb is that wherever there is such rate-based modulation tuning (bandpass “modulation transfer functions”) the neurons phase-lock to the envelope (modulation) fluctuations. The modulation spectrum also has correlates in spike timings, and consequently in autocorrelation functions in the form of longer time intervals (>10–20 ms). Because many neural subpopulations in the auditory pathway phase-lock to these slower periodicities, there are also likely to be temporal pattern coding correlates as well as those based on latencies.

More centrally, oscillatory phase-locked loop mechanisms similar to those proposed for somatosensory systems (sections 3.9 and 7) could potentially be triggered by onsets to enable oscillatory phase-offsets of spikes (spike latencies) to encode envelopes (Ahissar et al., 2001; Ghitza, 2011).

Time-domain representations and analysis of speech waveforms that emulate auditory phase-locked responses have proven effective. An acoustic processor for speech with single sample temporal resolution, that directly phase-locks to speech waveforms, demonstrated improved phonetic and sub-phonetic detections and segmentations, based on acoustic discontinuities (Baker et al., 1972; Baker et al., 1974; Baker, 1975). When integrated with standard spectral features in the HEAR acoustic processor, this time-domain processing improved test performance of a state-of-the-art continuous natural language speech recognition system on a standard corpus (Bahl et al., 1978; Baker, 1979).

Interval-place (Ghitza, 1992) and purely temporal, non-place representations (Ghitza, 1988) were explored as front-ends for speech recognition research systems. The non-place Ensemble Interval Histogram (EIH) representations are close to those population-interval distributions described above for pitch, albeit with some subtle differences that stem from their use of first-order rather than all-order intervals.

*Rhythm.* Because there are phase-locked responses to event onsets at all levels of the auditory system, rhythms in music (Nozaradan, 2014) and speech (Peelle and Davis, 2012; Peelle et al., 2013) have direct phase-locked temporal pattern encodings.

This is all in keeping with the general conjecture that if it’s in the waveform, periodic or not, it’s likely to be in the spikes. Provided that amplitude, frequency, and phase fluctuations lie within the frequency limits of phase-locking, it is likely that salient information will have direct correlates in the temporal patterns of spiking.

### 6.2 Sound localization

Temporal coding in binaural localization and echolocation, i.e., spatial hearing, is much more widely appreciated than its role in representing other auditory qualities. Spatial hearing includes estimating the direction (azimuth, altitude) of sound sources as well as their range (distance), size, and shape. It also includes the perception of sound fields in natural and architectural contexts such as concert halls, where time delays associated with reverberations are important (Ando, 2009). Even the psychophysical and neurophysiological literature addressing timing issues for spatial hearing, including binaural localization and bat and cetacean echolocation is rather large, but there are a number of excellent overviews and computational models (Carr et al., 2001; Grothe et al., 2010; Ming et al., 2021; Casseday and Covey, 1995; Simmons et al., 2003; Simmons et al., 1993; Simmons and Ferragamo, 1993; Durlach and Colburn, 1978; Colburn, 1996; Simmons et al., 1996b; Ming et al., 2021).

*Binaural sound localization*. Humans can localize sound sources in the horizontal plane with surprisingly high acuity. The two major cues are interaural time differences (ITD’s) and interaural level differences (ILD’s). When sounds propagate from some angle off the listener’s midline, the sounds arrive at the two ears at slightly different times, the difference being the ITD. Maximum ITDs in humans range from roughly 0–500 microseconds, depending on head size, such that this cue is operant for pure tone frequencies up to 1.5–2 kHz, above which ILD cues caused by acoustic head shadows are more effective. Lower frequency modulations (envelopes) of higher frequency carriers can also use ITD cues. The best jnd’s of 1–2 degrees azimuth correspond to interaural time differences of 10–20 microseconds (Grothe et al., 2010), which are comparable to time differences for best monaural pitch acuities (0.1–0.2% for 1 kHz pure tones).

Classically, the Jeffress Model has been the mainstay of ITD-based binaural localization computational mechanisms (Colburn, 1996; Cariani, 2011). It is a time-delay neural network consisting of (1) phase-locked spike train inputs from corresponding characteristic frequency regions in the two auditory nerves and ventral cochlear nuclei, (2) axonal tapped delay lines with different lengths and conduction times that convey the spike trains to (3) bipolar binaural neural coincidence detectors in the auditory brainstem that fire when spike from both left and right pathways arrive at the same time, and (4) neural coincidence counters whose firing rates provide a rate-place profile of ITDs.

Binaural localization in barn owls has been intensively studied because of their impressive hunting skills in almost total darkness (Grothe, 2018). They have excellent phase-locking limits (∼10 kHz) that are about an octave above ours and neural coincidence detectors that minimize jitter. By comparison, despite their much smaller head size, barn owls have extremely good localization with 4 degree minimal audible angles (Krumm et al., 2019). There are differences in binaural cross-correlation circuitries and topographic organization of ITD maps between mammals and birds (Carr and Konishi, 1990; Carr, 1993a; McAlpine and Grothe, 2003), such that the bird binaural circuits better match the details of the Jeffress Model than do those of mammals (Grothe, 2003; Grothe et al., 2010). In recent decades, roles for precisely-timed inhibition in sharpening coincidence detection have been incorporated into binaural models in order to account for neuroanatomical and neurophysiological differences between mammals, birds, and other animals (Grothe et al., 2010; Ashida and Carr, 2011).

Those implementation details notwithstanding, there are pervasive commonalities between time-delay cross-correlation mechanisms that compute very fine temporal disparities. Across the animal kingdom analogous temporal operations and mechanisms that measure time-of-arrival differences exist in many different modalities (Carr, 1993b): binaural localization, echolocation, somatic localization, electroreception, as well as gustatory and olfactory localization/lateralization (von Békésy, 1964b,c; Bower, 1974; von Békésy, 1967). From his interest in binaural and somatic localization based on different times-of-arrival at body locations, von Bekesy delivered tastants to different sides of the tongues of human subjects who were able to discriminate temporal orders down to a millisecond (von Békésy, 1964a,c). He carried out analogous experiments with olfactory localizations using air puffs into the two nostrils.

In binaural localization, the ridges on pinnae create spectral notches that can serve as “pinna cues” for sound direction in the vertical plane. Traditionally these cues have been assumed to be coded by rate-place profiles, but because every spectral feature has a corresponding time-domain correlate, these ridges also produce low-frequency envelope modulations that in turn produce phase-locked spike timing patterns in auditory nerves. Thus localizations using pinna cues might also be temporally-coded (Alves-Pinto et al., 2014).

*Echolocation.* Echolocation uses sounds emitted by a human or animal and their echoes in order to determine the presence, distance, and in some cases, shapes of external objects around them. It is especially useful in dark environments where vision is highly limited. The best echolocators are bats and cetaceans (Popper and Fay, 1995; Surlykke et al., 2014; Ladegaard et al., 2019; Ming et al., 2021) that form auditory images of the spaces and objects around them.

For pragmatic reasons, much more is known about the details of bat echolocation and its neuroanatomical and neurophysiological substrates than those of cetaceans (Casseday and Covey, 1995; Moss and Schnitzler, 1995; Covey, 2005). Despite very different environments and mediums, there are many computational commonalities between them. Both systems are capable of exploiting sub-microsecond temporal disparities.

The Spectrogram Correlation and Transformation (SCAT) model of Simmons and co-workers can be applied to both bat and cetacean echolocation (Simmons et al., 1996b; Ming et al., 2021). What follows is a highly simplified summary of the signals and signal processing computations.

Bats tend to use high frequency-swept chirps, whereas cetaceans tend to use high-frequency pulsatile clicks. Both acoustic bursts are very short. When a sound is emitted, if its echo copy is heard after some time delay, then there is an object somewhere in the direction of the sound source. The delay provides a readout of the distance to the object that depends on the velocity of sound in the medium (331 m/s in dry air or 1500 m/s in water).

The neural coding of both the vocalization and its echo produces a precisely timed spike at each characteristic frequency channel in the animal’s auditory nerve. In each channel, this produces an interspike interval that encodes the echo-delay. Depending on the shapes of objects, which are at different distances, different frequency channels can have slightly different echo-delays (glints) and by comparing intervals across channels, object variations in depth can be sensed. If the temporal cross-correlations are integrated with built up binaural representations then (2- and 3-D shape) contours can be inferred (Simmons, 1996; Simmons et al., 1996a). Narrow acoustic beams can act like searchlights, thereby simplifying the problem of interfering echoes (acoustic clutter).

## 7 Mechanoreception

Mechanoreception, mechanosensation, or the tactile sense encompasses sensations related to mechanical movement of the skin. Flutter-vibration is the sensation that is analogous to auditory pitch, and like pitch, there is strong evidential basis for phase-locked temporal pattern coding (Werner and Mountcastle, 1965; Mountcastle, 1967; Keidel, 1968; Mountcastle et al., 1969; Mountcastle et al., 1990; Johnson and Hsiao, 1992; Harvey et al., 2013). (Mountcastle, 1988) is an excellent starting point.

Humans can distinguish mechanical vibratory periodicities over a wide range of frequencies, from 5 to 300 Hz and above, depending on stimulating conditions (Talbot et al., 1968; Keidel, 1984; Morley et al., 1990). Flutter-vibration sensations can also be elicited by different frequencies of electrical microstimulation of the hand, up to 1000 Hz (Mountcastle, 1993). This is consistent with localizations on the skin and tongue based on down to 1 ms differences in electrical pulse arrival times (von Békésy, 1967). He carried out analogous experiments with non-electric olfactory and gustatory localizations used air puffs into the two nostrils and injected solutions of tastants onto the tongue, with comparable results (von Békésy, 1964c; von Békésy, 1964a).

Two classes of sensory neurons that innervate mammalian skin respond to vibratory stimuli. These are rapidly adapting (RA) fibers that innervate Meissner corpuscle end-organ receptors that are sensitive to light touch and slip on the skin. RA fibers phase-lock in the 5–100 Hz range and those innervating Pacinian corpuscle receptors, which phase-lock in the 30–1000 Hz range. An alternative theory based on ratios of firing rates of the two fiber types was inconsistent with results of experiments using 30 and 150 Hz vibrotactile stimuli with different amplitudes (Morley and Rowe, 1990). Because of phase-locking, complex tactile sequences, such as reading Braille, will have complex temporal pattern correlates in spike trains (Morley et al., 1990). Roughness in tactile perception would also presumably have similar correlates in phase-locked time patterns, but see (Johnson and Hsiao, 1992) for another view.

Mountcastle found that while first-order intervals are successively disrupted by accumulating jitter and intervening spikes as one goes up the somatosensory pathway, phase-locked all-order interval patterns of the vibratory stimulus were maintained all the way up, from primary sensory fibers to cortex (Mountcastle, 1993). Thus, like the auditory coding of pitch, the code for vibration pitch appears to be based on all-order intervals.

A recent study found evidence for millisecond-precision temporal coding in macaque somatosensory cortex (S1) of vibrotactile pitch for low frequencies (20–100 Hz) (Callier et al., 2024). Other studies have found mixtures of rate and temporal codes. Other studies of vibrotactile responses in rapidly-adapting fibers innervating glabrous skin of rats and mice found evidence for both rate and temporal codes (Medlock et al., 2024). Recent evidence in mice (Prsa et al., 2021; Lee et al., 2024) based on probability of phase-locking supports a temporal code for vibrotactile pitch (100–1900 Hz) at subcortical levels that is transformed to a rate code at the thalamus. Another study in S1 cortex in rats found rate and temporal codes for stimulus location and random vibrational waveform patterns (Blanc and Coq, 2007). Similar mixtures of multiplexed rate and temporal codes have been proposed for perception of spatial edges on the skin (Lankarany et al., 2019).

A great deal of work has been carried out on active sensing in rodent vibrissal systems by Ahissar and Vaadia (1990) (see section “3.9 Discussion”). They regard vibrissal systems as active sensing systems par excellence. Early on they found evidence for phase-locking and spike latency coding in the somatosensory pathway, as well as units with oscillatory characteristics in somatosensory cortex (Ahissar and Vaadia, 1990), spike latency-to-firing rate transformations (Ahissar et al., 2000), different sets of temporal codes for active and passive processes that can also account for vibrissal hyperacuity (Szwed et al., 2003; Knutsen and Ahissar, 2009).

Coding of sensory information gleaned by whiskers concerning coarseness of surfaces is related to vibration roughness perception in the skin (Arabzadeh et al., 2006), and here too, temporal codes can convey information related to whisker deflections (Jones et al., 2004; Jadhav et al., 2009). In the rat barrel cortex, first spike latencies may “form the basis for a fast robust population code” (Petersen and Diamond, 2000; Petersen et al., 2002b,2002a).

As with other modalities, these mechanoreceptive systems bear structural and functional similarities to other sensory systems in vertebrates and invertebrates. These similarities involve similar types of receptors (Itō, 1992), phase-locked responses to temporal onsets and patterns, e.g., as in insect cercal systems (Aldworth et al., 2011), as well as common active sensing mechanisms (Ahissar et al., 2023).

## 8 Electroreception

Weakly-electric fish, gymnotoform mormyrids such as knifefish and elephantnose fish, generate weak alternating sinusoidal electric fields for localizing prey and for intraspecies communications (Bullock, 1982). By means of electroreceptors that detect changes in electrical fields, and highly precise phase-locking of spikes, they can sense subtle distortions in the fields that are caused by other animals in their immediate vicinity. This electroreceptive sense is useful in detecting prey in turbid water. The distortions cause patterns of different latencies in neural spiking at different places on their body surfaces that indicate the direction of nearby prey.

These electroreceptive sensory systems can be regarded as phase-locked spike latency codes that have similarities to both binaural and somatic localization and echolocation. The latencies can be regarded as relative delays or alternately as different phase or time offsets in relation to sinusoidal electric fields and electric pulses, respectively (Heiligenberg, 1989; Heiligenberg, 1991; Heiligenberg, 1994). The system thus resembles the temporal auto-correlation and cross-correlation computations that are used by other modalities (binaural hearing, echolocation, somatic, gustatory, and olfactory localizations, (von Békésy, 1967; Carr and Soares, 2002) in vertebrates and insects.

Specialized receptors, gap junction electrical synapses, delay lines, and neural coincidence detectors reduce spike timing jitters, enabling the fish to detect sub-microsecond time differences (Carr et al., 1986). Thus, in the electroreceptive system a latency-place code appears to be converted to a channel code in which various combinations of body points are represented. As with the Jeffress delay-coincidence architectures, the output need not necessarily be a rate-channel code at higher levels of the system. For example, the next stage of processing could be a latency-place code rather than another rate-channel code.

The fish also use their generated sinusoidal fields to communicate with each other (Hopkins, 1988). The fields are regular, very nearly sinusoidal, and typically have frequencies of several hundred Hz. As with bats and cetaceans, to avoid jamming, the fish adjust the signals they produce based on sensed sub-millisecond timing differences (Baker et al., 2013). And as in human speech and animal vocalizations, multiple kinds of temporally-coded information are multiplexed together in communications signals.

## 9 Vision

### 9.1 Form vision

Historically vision has largely been envisioned as a rate-channel sensory modality in which time plays little or no role. However, coming from the perspective of the auditory neurogram and interspike interval distributions of Figure 2 and the visual interspike interval distributions of Figure 3, it would appear that the neural coding at its earliest stages in the retina, optic nerve, and thalamus (lateral geniculate body, LGN) may be a phase-locked spatiotemporal pattern code, with visual forms being spatial patterns of near-synchronous, phase-locked spikes. In the figure, the period and interval histograms show clear phase-locking (spikes and intervals under the line) to the sinusoidal temporally modulated constant velocity moving grating. Phase-locking can also generate different, but reliable first spike latencies, if retinal elements fire at different phases and relative times. Reliable relative latencies in retinas and higher centers can encode contrasts with high temporal precisions (Pollen et al., 1989; Bialek et al., 1991; Reinagel and Reid, 2000; Gollisch and Meister, 2008; Baden et al., 2011; Gutig et al., 2013). If spike response jitters of ∼1 ms or less are present (Carney et al., 1995), then the unexpected vernier acuities observed for high velocity moving targets may be explicable in terms of phase-locked millisecond-precise spike timings. See discussion of the hyperacuity problem above (section 4.6).

As in relational, correlation-based theories of visual form (Kabrisky, 1967; Uttal, 1987, 1988) and texture (Uttal, 1975), auto- and cross-correlation of spatial intervals and phase relations, respectively, would enable representations of patterns of edges and even points (dotted forms). Similar in many respects to temporal correlation-based scanning models (Reitboeck et al., 1988; Pabst et al., 1989; Reitboeck, 1989), lateral delay lines and coincidence detectors would convert spatial intervals and phase-relations into temporal, all-order interspike intervals, as in Figure 3A, and relative spike time-of-arrival patterns that would encode phase relations. For critical perspectives and debates on the role of eye movements in vision, see (Ahissar and Arieli, 2012; Rucci et al., 2018; Rucci et al., 2025), and (Gur, 2024).

This kind of spatiotemporal representation based on phase-locking at its root would seem to be consistent with the necessity of image motion or flashed transients for form vision. Forms rapidly disappear when images are stabilized (Ditchburn and Ginsborg, 1952), in as little as tens of milliseconds (Coppola and Purves, 1996). When images are well-stabilized, phase-locking should be completely abolished. As with the order-of-firing codes discussed above (section 4.6), quick encoding of images would only require one-spike-per-channel in only a fraction of channels (Gautrais and Thorpe, 1998; Van Rullen et al., 2005). This is also consistent with form-from-temporal-synchrony visual percepts, a.k.a. “illusions” (Lee and Blake, 1999b,a; Blake and Lee, 2005), and the role of interocular latency timing disparities, presumably reflected in phase-locked spike times, that the visual system interprets as depth cues in the Pulfrich Effect (Graham, 1966; Lanska et al., 2015).

There are many parallels between auditory and visual representations and Gestaltist grouping principles (Ando, 2009). There is a perception of a visual flicker stimulus at the “missing fundamental” of harmonically-related flicker components (e.g., one can match a combination of 4, 5, and 6 Hz flickers to a 1 Hz flicker). There is also a spatial frequency analog wherein gratings of 4*f*, 5*f*, and 6*f* spatial frequencies can be matched to a grating with the fundamental frequency *f* (de Valois and de Valois, 1990).

Historically, most theories of vision have been cast in terms of rate-channel codes, receptive fields, and local feature detectors rather than in terms of correlated patterns of spikes. Uncontrolled eye movements and extra spikes complicate the detection of phase-locked spikes. Under-controlled stimulus timing also complicates detection of phase-locking. For example, due to uncertainties in the field phases of the monitor that was used to deliver the moving gratings in Figure 3A, periodicities higher than ∼16 Hz were smeared out in post-stimulus time (PST) histograms. Higher frequency patterns were only clearly resolved in the all-order interspike interval distributions shown. For the auditory interval histograms of Figure 2, interval histograms were compiled for each trial and then added together. If they look at interspike interval distributions, most vision researchers examine only first-order intervals to compute firing rates, in which periodicity patterns can be obliterated by intervening spikes (e.g., bursts, mixtures of phase-locked spikes with different latencies or non-phase-locked spikes). Standard vision theory has also not been helpful because it assumes, often tacitly, that any phase-locked spikes due to motion or transients is converted, via a layer of motion detectors, to rate-place codes, after which, according to the theory, all fine timing related to phase-locking can then be ignored.

All the difficulties notwithstanding, there have been many visual neurophysiologists who have looked hard for visual information in fine spike timing cues and their correlations (Victor and Purpura, 1996). Temporal precisions of spikes increase for changing (moving or fluctuating) images, as opposed to static ones (Mechler et al., 1998). Temporal codes appear to be more stable (reliable, robust) than their rate-based counterparts (Zhu et al., 2025) as well as being more efficient (Price and Gavornik, 2022).

There were earlier proposals for temporal coding (Reichardt, 1961), multiplexing (Chung et al., 1970; Wasserman, 1992) and correlation-based theories of brain function (von der Malsburg, 1994), but interest in these issues really blossomed in the 1990’s. There is a sizable literature from that period concerning visual codes and operations: synchronies (Singer, 1994b,a; Konig et al., 1995; Singer, 1999), oscillations (Gray et al., 1989; Gray et al., 1992), spike correlations (Mastronarde, 1989; Lestienne et al., 1990; Abeles, 1994; Lestienne, 1996; Lestienne and Tuckwell, 1998; Gerstein, 2004; Abeles, 2009), temporal coding (Bialek et al., 1991; Egelhaaf and Borst, 1993; Gawne, 1999; Oram et al., 1999), multiplexing (Richmond et al., 1989; Richmond et al., 1990; McClurkin et al., 1991a; McClurkin et al., 1991b; McClurkin et al., 1991c; Eskandar et al., 1992; Eskandar et al., 1992; McClurkin et al., 1993; McClurkin and Optican, 1996; McClurkin et al., 1996), and feature-binding (Eckhorn and Reitboeck, 1990; Eckhorn, 1991; Singer and Gray, 1995; Gray, 1999).

However, it is not clear to us where the field went and where it currently stands, e.g., encoding of spatial frequencies in gamma rhythms (Han et al., 2021) and distributed processing via temporal-channel codes (Singer, 2009, 2018). It seems that these as well as other fundamental questions involving the neural codes for basic visual attributes such as form, texture, and color still remain to be solved.

### 9.2 Visual texture

In the auditory system, pitches and timbres of resolved harmonics are famously insensitive to differences in phase-spectra (envelopes of unresolved harmonics are a different matter). The AM and QFM tones in Figure 2 are perceptually indistinguishable, despite quite obvious differences in their waveforms. This is why an inherently phase-insensitive autocorrelation-like all-order interval code is necessary to account for auditory percepts.

Visual texture representations appear to be analogously insensitive to phase – they cannot be pre-attentively distinguished (Pabst et al., 1989). Their discrimination is dependent only on spatial interval statistics as in spatial autocorrelations and not on phase information, as in image cross-correlations (Kabrisky, 1967; Uttal, 1975, 1987, 1988). Autocorrelation representations are sensitive to frequency content and disparities but insensitive to phase disparities, whereas cross-correlations of waveforms are highly sensitive to phase disparities (e.g., the AM vs. QFM waveforms of Figure 2). If spatial intervals are converted to time intervals in the visual system, as the scanning models do (Reitboeck et al., 1988; Pabst et al., 1989; Reitboeck, 1989), then auditory timbre and visual texture perception would have many coding similarities. Roughness also has analogs in both auditory and visual textural domains (Ando, 2009).

Information concerning visual textures can be found in spike timings (Pollen et al., 1985; Victor and Conte, 1996). Characteristic texture percepts can also be reliably induced by particular temporal patterns of flicker (Wilson, 1960; Fiorentini and MacKay, 1965; Perkell and Bullock, 1968; Young et al., 1975; Richmond et al., 1989). Retina and visual cortex contain many slow and fast horizontal delay paths that generate two-dimensional standing wave spatial patterns of excitations when rhythmic flicker stimuli are presented (Siegel and Read, 1993) that are perhaps not unlike those created by spatiotemporal patterns of neural responses to regular visual textures. Of related interest were rather striking subjective responses that were sometimes evoked by flashed visual stimuli delivered at particular phases of alpha rhythms (Walter, 1959; Hutchinson, 1991).

### 9.3 Color

Color may be a temporally-coded percept. These sensations have been variously called flicker colors, subjective colors, Prevost-Fechner-Benham subjective colors, Fechner color, or pattern-induced flicker colors (PIFCs). In the early 19th century, the perceptual phenomenon was discovered first by a French monk Benedict Prevost and then by the psycophysicist GustavFechner that flickering white light can induce different color sensations. See (Cohen and Gordon, 1949) for a detailed early history. Fechner designed several black and white disks that, when rotated below the flicker fusion frequency limit, produced different colors depending on the relative durations of alternating black and white segments. Figure 3B shows disks constructed and investigated by Fechner, Helmholtz, and Benham, all of which produce a range of colors, depending on the rotational speed of the disk. In Benham (1894) created a top, called the artificial spectrum top, or Benham’s top, which was sold as a toy (Benham, 1894, 1895).

As a historical note, in the early 1960’s a shuttering device, the Butterfield Color Encoder, was built for black-and-white (B&W) television cameras that converted color images to B&W flicker patterns that could then be broadcast to B&W televisions whose viewers then, in many cases unexpectedly, saw color images on their B&W TV screens (Griffin, 1968; Sheppard, 1968).

Many years later Festinger, Allyn, and White, using glow tubes, determined the temporal patterns that evoke particular colors, Figure 3B (Festinger et al., 1971). All colors normally seen by trichromat humans can be produced this way. The temporal patterns have been Fourier-analyzed (Tritsch, 1992).

On its face, the Prevost-Fechner-Benham Effect appears to strongly suggest a neural code for color (Sheppard, 1968). Many different classes of retinal cells phase-lock to the coarse temporal monochromatic flicker patterns. If the central neural code for color is a temporal pattern of spikes, then the central visual system would interpret the flicker patterns as color information. When the eye is presented with a visual scene with different mixtures of wavelengths, different activation latencies and lateral inhibitory interactions might naturally produce these patterns.

There has been a good deal of debate over the years about whether the flicker patterns might have differential effects on different classes of cones due to lateral inhibition or different temporal response properties, e.g., (von Campenhausen, 1969; von Campenhausen et al., 1992), but these hypotheses are not necessarily inconsistent with a temporal code for color, as they might be the means by which the system encodes color information. However rate-channel coding of specific subpopulations would appear to be falsified by electrical stimulation experiments.

Electrical stimulation of the whole eye in human subjects using the time patterns of Festinger et al. (1971) produced phosphene flashes of colors similar to those evoked by the glow tube (Young, 1977). Since electrical stimulation presumably excites all retinal cells to fire in the same temporal pattern, this is appears to be strong evidence in favor of a central temporal code for color.

There is some evidence for characteristic temporal patterns in normal color vision. Such temporal patterns have been found in optic nerve (Kozak and Reitboeck, 1974; Kozak et al., 1989), lateral geniculate (Young and De Valois, 1977), and visual cortex (Richmond et al., 1989; McClurkin et al., 1993). A model for the representation of color using temporal encoding machines (TEMs, section “13 Design of artificial systems”) to separate multiple temporal spike pattern components has been proposed (Lazar et al., 2015).

## 10 Chemical senses: gustation and olfaction

Because phase-locking cannot track the vibratory frequencies of molecules, phase-locked temporal pattern codes are ruled out. However, stimulus-triggered temporal pattern codes, spike latency codes, and mixed latency-channel codes are all still possible. Because electrical stimulation of whole populations appears to mimic chemical stimulation in the gustatory system, a temporal pattern code appears most likely for gustation. On the other hand, due to differences in latencies of its receptor and neuronal responses as well as some channel specificities, spike latency and latency-channel codes appear to be the most likely types of temporal codes to be operant in olfaction. In both systems, temporal and rate-place codes can coexist, both in the same neural populations and in separate ones.

### 10.1 Gustation

Strong evidence exists in favor of temporal pattern codes for taste in the gustatory pathways of vertebrates and arthropods (Katz, 2005; Glendinning et al., 2006; Hallock and Di Lorenzo, 2006; Di Lorenzo et al., 2009; Ohla et al., 2019). Often both temporal and rate codes are found together, sparking active discussions and debates about temporal vs. rate coding vs. multiple codes in specific neural populations (Spector and Travers, 2005; Wilson et al., 2012; Jezzini et al., 2013; Reiter et al., 2015; Staszko et al., 2020; Roper, 2022).

Through most of the history of modern neuroscience, following (Adrian, 1928), it was assumed that taste buds (gustatory papillae) responded only to specific tastant classes. Consequently, gustatory sensations were thought to be determined entirely by firing rates in the primary neurons of the gustatory system, in the chorda tympani. However, doubt was cast on these assumptions when highly specific taste receptors were not found (Kimura and Beidler, 1961; von Békésy, 1964c). Early and subsequent single-unit neurophysiological experiments began to find temporally patterned responses.

Experiments with electrical stimulation of the tongue to produce taste sensations began at the dawn of electrophysiology, first being “described by Sulzer, a few decades before Volta’s famous experiments”(von Békésy, 1964c, i.e., ca. 1752–1754). von Békésy (1964d) carried out a series of electrical stimulation at various tongue locations and temporal frequencies and was able to elicit sensations of what are thought to be the four primary taste classes: sweet, sour, bitter, and salty.

In Covey (1980) Covey recorded whole chorda tympani responses to four taste classes presented to decerebrate rats (Figure 3C). When the tastants are presented, the animals, despite the decerebration, elicit mouth gestures that are similar to those of intact rats and that are characteristic of tasting sweet, sour, salty, or bitter flavors. Stimulation of the chorda tympani with the same temporal patterns, but not with other patterns, elicited the corresponding behavioral gestures. In the decade following, Patricia DiLorenzo carefully replicated Covey’s experiments and carried out similar ones in the nucleus of the solitary tract, the next station in the ascending gustatory pathway.

In, Di Lorenzo and Hecht (1993) summarized the early situation:

“In the study of the neural code for gustation in the central nervous system, the temporal patterns of responses to taste are most often ignored. Typical measures of taste responses account for the overall amount of neural activity evoked by a tastant but do not reflect the temporal arrangement of spikes during the response. These measures would be adequate descriptors if the total number of spikes associated with a given response were equally distributed within the response interval; however, that is almost never the case. Instead, most taste responses are characterized by variations in the rate of firing. The time course and magnitude of these variations defines the temporal pattern of a response. Given numerous reports that different taste stimuli appear to evoke distinctive temporal patterns of response in a number of taste-related neural structures and that similar-tasting stimuli evoke similar temporal patterns of response [15 citations ranging from 1957 to 1989, omitted here] it is not surprising that several investigators have suggested that this feature of the neural response may contain important, if not essential, information about taste stimuli.”

For more than four decades now Patricia DiLorenzo and co-workers have expanded this line of investigation into temporal coding of gustation to deepen understanding of the neuroanatomy and neurophysiology of gustatory systems and their functioning: (Di Lorenzo and Schwartzbaum, 1982; Di Lorenzo, 1989, 2000; Hallock and Di Lorenzo, 2006; Di Lorenzo et al., 2009; Di Lorenzo, 2021).

### 10.2 Olfaction

Strong evidence exists for spatiotemporal, latency-channel and firing sequence codes in olfaction Chong and Rinberg, 2018; Chong et al., 2020; Haddad et al., 2010; Haddad et al., 2013; Chong and Rinberg, 2018; Perl et al., 2020; Verhagen et al., 2023; Chong et al., 2020). See (Wang et al., 2024; Uchida et al., 2014) for excellent reviews.

Complex rate-coded temporal patterns have historically been observed, and these have baffled olfactory neurophysiologists (Lettvin and Gesteland, 1965; Gesteland et al., 1968). In part this has been due to the complexity of the sense of smell, where the dimensionality of the perceptual space has been a matter of dispute. This is because there are on the order of 1000 different receptor types in the human olfactory system, such that assumptions of channel coding of odor identity would require a similar number of dimensions. Adding to the problem is the lack of any clear low-dimensional structure to chemical odorant space (Haddad et al., 2008), unlike a cochleotopic frequency map in audition or a spatial retinotopic map in vision. It is difficult to identify what the primaries of the space would be (Boring, 1942). Rather than a trillion discriminable odor combinations that suggest a high-dimensional space, the number of dimensions and distinctions may be much smaller and more manageable (Meister, 2015). There are some opponency relations, where pairs of odors can cancel each other out. Still more challenging is the problem of stimulus invariance with respect to intensity, here odorant concentration. Some odorants elicit different percepts at different concentrations. Finally, receptors in the olfactory bulb are constantly turning over every few weeks, such that the system is constantly being rewired, potentially complicating labeled line and channel-based coding and network learning strategies.

A stable set of temporal codes might solve this problem of coding constancy, and investigators have found temporal pattern correlates of some odorant types in the past (Macrides and Chorover, 1972; Macrides, 1977; Meredith and Moulton, 1978; Meredith, 1981; Kauer, 1990; Kauer and White, 2001). Inspired by different latencies of response to odorants, (White and Kauer, 2001), Kauer, White, and co-workers developed an artificial nose using synthetic DNA-based receptors and similar coding principles (White et al., 2008).

There is also phase-locking to sniffing cycles (Mori and Sakano, 2022; Macrides and Chorover, 1972 #7592), which though not necessarily essential for coarse olfactory discriminations, may nevertheless improve odor discrimination acuity. Similarly, oscillations that emerge when engaging in active smelling (sniffing) may have functional relations to neural coding (Freeman, 1975; Kauer, 1998; Dorries and Kauer, 2000; Kauer, 2002; Kay et al., 2009).

Although the olfactory system has traditionally been regarded as operating over relatively long timescales (hundreds of milliseconds to seconds), rodents can make fine odorant discriminations and invariant recognitions despite rapid fluctuations of odorant concentrations on short timescales (10–30 ms). These fast changes yield temporally-coded signatures that could be used (1) for active sensing discounting of inhalation dynamics so as to realize odor quality/identity invariance (Jordan et al., 2018), (2) for gaining information about odor plumes and the environment to facilitate tracking of odors and separating out multiple odor plumes in natural environments (Ackels et al., 2021), and/or (3) separating out temporal neural response components related to odorant concentrations and their onset dynamics from other later components that are invariant with respect to odor quality and odorant identity (Lazar et al., 2023). Robotic olfactory systems have been designed and constructed to take advantage of fast olfactory processing and temporal coding (Dennler et al., 2024).

The research group of Giles Laurent and former co-workers has investigated temporal and correlation coding (Laurent et al., 1996; Laurent, 1999; Wehr and Laurent, 1999; Friedrich and Laurent, 2001; MacLeod and Laurent, 1996), temporal sequence codes (Wehr and Laurent, 1996), spatiotemporal codes (Laurent et al., 1998), oscillations (Perez-Orive et al., 2002), and dynamical system trajectories (Laurent, 2002; Mazor and Laurent, 2005) in the mushroom body of locusts. In an experiment to test the functional role of population oscillations in the locust mushroom body, they applied picrotoxin (Stopfer et al., 1997), which abolishes the oscillations, and found that fine, but not coarse, odorant discriminations were impaired. One interpretation is that oscillations facilitate better coding, but that they are not essential for making more basic distinctions. Another would be to postulate dual codes.

Optogenetic stimulation has been used effectively to investigate the relative contributions of temporal, spatial, and spatiotemporal codes in the rat olfactory bulb (Haddad et al., 2013; Chong and Rinberg, 2018; Chong et al., 2020). Using the optogenetic technique, researchers have been able to independently drive the rat olfactory bulb using recorded spatial and temporal patterns of neural responses to natural and specially designed odorants. They find that particular coarse spatiotemporal patterns of stimulation are most effective in predicting perceptual judgments of smell.

## 11 Cutaneous sensations (pain, itch, temperature)

The cutaneous senses typically include tactile sensations (touch, vibration, pressure), pain, itch, temperature, and proprioception (body position, joint positions, muscle stretch). Touch and vibration are covered in section “8 Electroreception”; proprioception and the vestibular sense in section “13 Design of artificial systems.”

The somatic senses of pain, itch, and temperature have mainly been approached from labeled line and overlapping neuronal populations. Ma (2010) provides a good review of the neural coding problem as it pertains to these senses. They note that “the activation of specific sensory fibers is sufficient to evoke a specific somatic sensation.” But inter-modality interactions, e.g., pain and itch, hot and cold, and touch and pain, are problematic for simple labeled line theories. In order to explain these interactions “population coding” theories in which there are separable neural populations that have some cross-talk have been proposed (Ma, 2010). Perhaps counter intuitively, different separate populations may be responsible for the sensations of hot and cold (Wang et al., 2018). Rate-based multiplexing of neural responses in rat S1 to itch and pain also are relevant to the cross-talk problem (Woo et al., 2022).

Another review (Bokiniec et al., 2018) takes up similar sensory crosstalk issues, but notes that there are proposed codes that “combine specialized receptors with temporal coding schemes.” Note that “temporal coding” in these contexts can mean coarsely-scaled temporal successions of activations of different populations of neurons rather than temporal patterns of spikes. Burst-pattern coding has been proposed for thermoreception, but it is not clear how well it would explain such multimodal cross-talk phenomena. See (Doetsch, 2000; Green, 2004; Perl, 2007; Prescott et al., 2014) for discussions of non-temporal labeled line, gate-control, channel-pattern codes, and cross-talk population codes.

Despite its importance and extensive clinical data, the neural codes that subserve sensations of itch and pain are still poorly understood (Schmelz, 2015, 2021). It appears that primary somatosensory nocioceptive afferents are multimodal, such that they respond to both pain and itch provoking stimuli (Ikoma et al., 2006; Hu et al., 2021). Different firing patterns have been hypothesized to encode the different sensations in the same neurons: burst-like activity for itch vs. single action potentials for pain (Sharif et al., 2020).

From a temporal coding perspective, by far the most intriguing experiments are those reported by Emmers (1966b),a, Emmers (1969, 1970, 1976, 1981). Recording in the thalamus, he found separate temporal pattern codes for different types of thalamic neurons sensitive to touch, pressure, thermal, gustatory, and pain stimuli (Emmers, 1981, p. 116). The spike patterns had the form of onset bursts of a few spikes followed by interspike intervals that were characteristic of the type of stimulus driving the unit. Some units showed temporally multiplexed and interleaved spike patterns. This work has largely been ignored we think because the results sound too good to be true, but these experiments really should be carefully replicated by others and their results, negative or positive, should be reported out.

## 12 Proprioception and movement

The proprioceptive system provides continuous feedback information about body movements. Stretch receptors phase-lock to both transient and periodic muscle movements, such that the somatosensory system receives a temporal pattern readout of all of the movements of muscles. Likewise vestibular afferents robustly phase-lock to acceleration transients (Curthoys et al., 2017), again providing temporal pattern feedback information for self- and externally-caused motion. In many cases, there also will be similar time patterns accompanying the self motion, in terms of changes in visual scenes correlated with head movements, and changes in acoustic scenes correlated with vocalizations. Given these commonalities of temporal structure in action and perception, temporal patterns of perceived events can inform their production. Likewise, temporal patternings used to produce a sequence of actions (such as a rhythmic drum sequence) can inform the perceptual detection and recognition of those patternings. For time patterns of perception and action, what goes around, comes around.

## 13 Design of artificial systems

Whereas science is primarily concerned with understanding how the natural world works using empirical data and models, engineering is ultimately focused on designing and constructing artificial systems that can perform useful functions. The two pursuits interact and mutually inform each other in the realms of computational neuroscience (Rieke et al., 1997) and neuromorphic engineering (Indiveri and Horiuchi, 2011; Indiveri et al., 2011; Kudithipudi et al., 2025). Computational neuroscience involves models of neuronal behavior and of how biological nervous systems achieve informational functions that guide behavior. Neuromorphic engineering involves the design and construction of artificial devices using principles from neuroscience that have been suggested by biological brains.

Despite their lack of many biophysical details, simplified engineering-inspired models of neural information processing can prove useful in considering putative coding and processing schemes in the brain. They can clearly show how some particular behavioral function might be achieved. Provided that their simplifications retain essential aspects of biological neural signals, elements, and architectures, these functional models can serve as demonstrations of principles.

Aforementioned examples of how temporally-coded inputs and putative subsequent signal processing can be used to realize various specific behavioral functions for sound localization (Jeffress Model, section “6.2 Sound localization”), pitch perception (Licklider duplex and triplex models section “6.1 Basic auditory qualities”), acoustic front ends (Baker’s HEAR and Ghitza’s Ensemble Interval Histogram (EIH), section “6.1 Basic auditory qualities”), artificial noses (section “10.2 Olfaction”), and active perception (phase-locked loops, section “3.9 Temporal coding in active sensing”).

Aside from brief discussions of central phase-locked loops for active perception (sections “3.9 Temporal coding in active sensing”, “11 Cutaneous sensations (pain, itch, temperature),” and “10.2 Olfaction”), a full treatment of how temporal sensory codes might be interpreted in central stations lies beyond the scope of this paper. Elsewhere we have proposed that brains may use multiplexed temporal codes and time-domain correlation-based operations for a much wider range of functions than generally envisioned, and have discussed the kinds of temporal processing neural architectures that might handle such codes (Cariani and Baker, 2022; Baker and Cariani, 2025).

Theories of how biological brains handle information can also provide new principles for designing more powerful and elegant artificial systems. Although most artifical signal processing, pattern recognition systems, neural networks, and robots have used channel-coded representational schemes, there is no reason that artificial computing and robotic systems cannot incorporate temporal coding as well. As observed in many sensory systems, temporal coding can provide more precise and robust representations of incoming sensory information than channel codes. Temporal codes also obviate the need for windows and their limitations. Early examples of temporal processing hardware architectures include analog (Fukushima et al., 1970) and analog VLSI implementations (Lazzaro and Mead, 1989; Mead, 1989; Douglas et al., 1995).

Although most neural networks to date have assumed scalar signals (artificial counterparts of firing rates), integrating elements, and sequences of coarse processing (time) steps, some neural networks have been proposed that explicitly incorporate fine time bases and individual spiking events. Examples include time-delay neural networks (TDNNs) (Tank and Hopfield, 1987), pulse-coded spiking neural networks (SNNs) (Gerstner, 1999; Maas, 1999; Sejnowski, 1999; Yamazaki et al., 2022), synfire chains and polychronous networks, and neural timing nets (Cariani, 2001c,^2004^).

A salient, widely recognized advantage of neural networks that process pulses (electronic and numerical analogs of spikes) is that they use much less power than comparable conventional multilayer neural networks. Although most spiking networks in the past have assumed pulse frequency modulation (PFM) encodings, i.e., rate-channel codes, temporal codes could also be used more widely in spiking neural networks of the future.

Address-event representations (AERs) (Srivatsav et al., 2023), event-based temporal coding and processing (Risi et al., 2020; Assa et al., 2025) and time encoding machines (TEMs) (Lazar and Pnevmatikakis, 2011; Lazar et al., 2015) import temporal coding strategies into artificial, pulse-coded devices for general computational signal processing (Indiveri and Horiuchi, 2011). As in the Fukushima et al. (1970) artificial retina, events consist of changes and their timings rather than static properties. These kinds of representations appear to enable continuous signals to be reconstructed from their pulse-coded event timings (Lazar and Tóth, 2004; Adam et al., 2020). Direct incorporation of temporal codes and time-domain operations into neural networks and other pulse-coded systems thus promises to be a fertile avenue for technological innovation in the future.

## 14 Conclusion

Evidence for temporal coding exists in virtually every sensory system, often coexisting with rate-channel codes.

In many sensory systems, spike precisions are on the order of a millisecond or less.

Temporal codes, as discussed here, can take the form of either characteristic temporal patterns of spikes or of characteristic patterns of spike response latencies.

The particular temporal spike patterns that are produced can be produced extrinsically, through phase-locking in which the stimulus impresses its time structure on response spike trains.

They can also be produced intrinsically, by the triggering of neurons and local circuits. The patterns produced reflect the internal organization of the neural circuits rather than the fine time structure of the stimulus.

First-spike latencies in response to onset events can be quite precise.

Combinations of temporal and channel-based codes are possible.

A number of sense modalities may rely on cross-channel patterns of first-spike latencies.

Firing sequence codes are temporal codes that rely on spiking order rather than metrical time relations.

Many auditory percepts related to stimulus qualities can be modeled in terms of auto-correlation-like interspike interval distributions.

Many auditory percepts related to localization may be explained in terms of temporal cross-correlations (direction) and auto-correlations (distance, echo-delay).

Early vision may turn out to rely on a phase-locked, spatiotemporal pattern code.

Phase-locked temporal pattern codes can use autocorrelation operations to represent the internal periodicities in stimulus waveforms. They can use temporal cross-correlations to represent different times-of-arrival of stimuli at different sensory surfaces and to analyze phase relations.

Further investigations into temporal coding promise a fertile ground for new scientific discoveries and technologies.

## Data Availability

The original contributions presented in this study are included in this article/supplementary material, further inquiries can be directed to the corresponding author.
